# Genetics of immune response to Epstein-Barr virus: prospects for multiple sclerosis pathogenesis

**DOI:** 10.1093/brain/awae110

**Published:** 2024-04-17

**Authors:** Jesse Huang, Katarina Tengvall, Izaura Bomfim Lima, Anna Karin Hedström, Julia Butt, Nicole Brenner, Alexandra Gyllenberg, Pernilla Stridh, Mohsen Khademi, Ingemar Ernberg, Faiez Al Nimer, Ali Manouchehrinia, Jan Hillert, Lars Alfredsson, Oluf Andersen, Peter Sundström, Tim Waterboer, Tomas Olsson, Ingrid Kockum

**Affiliations:** Department of Clinical Neuroscience, Karolinska Institutet, SE-171 77 Stockholm, Sweden; Centrum for Molecular Medicine, Karolinska University Hospital, SE-171 76 Stockholm, Sweden; Department of Clinical Neuroscience, Karolinska Institutet, SE-171 77 Stockholm, Sweden; Centrum for Molecular Medicine, Karolinska University Hospital, SE-171 76 Stockholm, Sweden; Science for Life Laboratory, Department of Medical Biochemistry and Microbiology, Uppsala University, SE 751 23 Uppsala, Sweden; Department of Clinical Neuroscience, Karolinska Institutet, SE-171 77 Stockholm, Sweden; Centrum for Molecular Medicine, Karolinska University Hospital, SE-171 76 Stockholm, Sweden; Department of Clinical Neuroscience, Karolinska Institutet, SE-171 77 Stockholm, Sweden; Infections and Cancer Epidemiology, German Cancer Research Center (DKFZ), DE-69120 Heidelberg, Germany; Infections and Cancer Epidemiology, German Cancer Research Center (DKFZ), DE-69120 Heidelberg, Germany; Department of Clinical Neuroscience, Karolinska Institutet, SE-171 77 Stockholm, Sweden; Centrum for Molecular Medicine, Karolinska University Hospital, SE-171 76 Stockholm, Sweden; Department of Clinical Neuroscience, Karolinska Institutet, SE-171 77 Stockholm, Sweden; Centrum for Molecular Medicine, Karolinska University Hospital, SE-171 76 Stockholm, Sweden; Department of Clinical Neuroscience, Karolinska Institutet, SE-171 77 Stockholm, Sweden; Centrum for Molecular Medicine, Karolinska University Hospital, SE-171 76 Stockholm, Sweden; Department of Microbiology, Tumor and Cell Biology, Karolinska Institutet, SE-171 77 Stockholm, Sweden; Department of Clinical Neuroscience, Karolinska Institutet, SE-171 77 Stockholm, Sweden; Centrum for Molecular Medicine, Karolinska University Hospital, SE-171 76 Stockholm, Sweden; Department of Clinical Neuroscience, Karolinska Institutet, SE-171 77 Stockholm, Sweden; Centrum for Molecular Medicine, Karolinska University Hospital, SE-171 76 Stockholm, Sweden; Department of Clinical Neuroscience, Karolinska Institutet, SE-171 77 Stockholm, Sweden; Institute of Environmental Medicine, Karolinska Institutet, SE-171 77 Stockholm, Sweden; Centre for Occupational and Environmental Medicine, Stockholm County Council, SE-171 77 Stockholm, Sweden; Department of Clinical Neuroscience, Institute of Neuroscience and Physiology, the Sahlgrenska Academy, University of Gothenburg, SE-413 45 Gothenburgh, Sweden; Department of Clinical Science, Neurosciences, Umeå University, SE-901 85 Umeå, Sweden; Infections and Cancer Epidemiology, German Cancer Research Center (DKFZ), DE-69120 Heidelberg, Germany; Department of Clinical Neuroscience, Karolinska Institutet, SE-171 77 Stockholm, Sweden; Centrum for Molecular Medicine, Karolinska University Hospital, SE-171 76 Stockholm, Sweden; Department of Clinical Neuroscience, Karolinska Institutet, SE-171 77 Stockholm, Sweden; Centrum for Molecular Medicine, Karolinska University Hospital, SE-171 76 Stockholm, Sweden

**Keywords:** EBV, EBNA1, HLA, GWAS, DRB1

## Abstract

Epstein-Barr virus (EBV) infection has been advocated as a prerequisite for developing multiple sclerosis (MS) and possibly the propagation of the disease. However, the precise mechanisms for such influences are still unclear. A large-scale study investigating the host genetics of EBV serology and related clinical manifestations, such as infectious mononucleosis (IM), may help us better understand the role of EBV in MS pathogenesis. This study evaluates the host genetic factors that influence serological response against EBV and history of IM and cross-evaluates them with MS risk and genetic susceptibility in the Swedish population.

Plasma IgG antibody levels against EBV nuclear antigen-1 [EBNA-1, truncated = amino acids (aa) (325–641), peptide = aa(385–420)] and viral capsid antigen p18 (VCAp18) were measured using bead-based multiplex serology for 8744 MS cases and 7229 population-matched control subjects. The MS risk association for high/low EBV antibody levels and history of IM was compared to relevant clinical measures along with sex, age at sampling, and associated HLA allele variants. Genome-wide and HLA allele association analyses were also performed to identify genetic risk factors for EBV antibody response and IM history.

Higher antibody levels against VCAp18 [odds ratio (OR) = 1.74, 95% confidence interval (CI) = 1.60–1.88] and EBNA-1, particularly the peptide (OR = 3.13, 95% CI = 2.93–3.35), were associated with an increased risk for MS. The risk increased with higher anti-EBNA-1 IgG levels up to 12× the reference risk. We also identified several independent HLA haplotypes associated with EBV serology overlapping with known MS risk alleles (e.g. DRB1*15:01). Although there were several candidates, no variants outside the HLA region reached genome-wide significance. Cumulative HLA risk for anti-EBNA-1 IgG levels, particularly the peptide fragment, was strongly associated with MS. In contrast, the genetic risk for high anti-VCAp18 IgG levels was not as strongly associated with MS risk. IM history was not associated with class II HLA genes but negatively associated with A*02:01, which is protective against MS.

Our findings emphasize that the risk association between anti-EBNA-1 IgG levels and MS may be partly due to overlapping HLA associations. Additionally, the increasing MS risk with increasing anti-EBNA-1 levels would be consistent with a pathogenic role of the EBNA-1 immune response, perhaps through molecular mimicry. Given that high anti-EBNA-1 antibodies may reflect a poorly controlled T-cell defence against the virus, our findings would be consistent with DRB1*15:01 being a poor class II antigen in the immune defence against EBV. Last, the difference in genetic control of IM supports the independent roles of EBNA-1 and IM in MS susceptibility.

## Teaser

Huang *et al.* examine how a person’s genetic make-up affects their immune response to Epstein-Barr virus and how this relationship affects risk for multiple sclerosis. Higher levels of Epstein-Barr virus antibodies are associated with increased multiple sclerosis risk, partly due to overlapping HLA associations.

## Introduction

Epstein-Barr virus (EBV) is a highly prevalent and persistent human herpes virus that can cause infectious mononucleosis (IM) and certain lymphoproliferative diseases.^1^ Infection typically occurs during early childhood, after which the virus remains latent in B-lymphocytes. Although persisting infections are mostly asymptomatic, viral reactivation can often occur when immunocompromised, as evident by the increased incidence of EBV-associated Hodgkin’s and Burkitt’s lymphoma with HIV-induced immunodeficiency.^2^ Many epidemiological studies have also implicated a consistent, yet often mechanistically unclear, role of EBV in immune dysfunction and risk of autoimmune diseases.^1,3,4^

Multiple sclerosis (MS) is an inflammatory disease characterized by the infiltration of peripheral immune cells into the CNS, resulting in myelin damage and subsequent injury to neurons and axons. Although the cause of MS remains elusive, characteristics of EBV infection, such as seropositivity and IM, are well established risk factors for MS.^4,5^ The serological response against EBV, particularly the nuclear antigen (EBNA), is associated with an increased risk of MS^6-8^ and disease progression (e.g. relapse rate, severity and the number of lesions).^9^ Recent evidence strongly suggests that EBV infection is a prerequisite for developing MS.^5,10^

However, identifying the direct mechanism remains a challenge and prevailing discussions have debated whether EBV response is a parallel phenomenon, perhaps due to overlapping genetic predisposition, or indicative of a direct causal role, either by the infection itself or the immune response against the virus. Several retrospective studies have shown EBV seroconversion and increased antibody titres among individuals several years before MS onset.^5,7,8,10,11^ Furthermore, genome-wide association studies have improved our understanding of EBV’s role in host immune regulation,^12,13^ indicating a possible causal link between EBV serology and MS,^12^ although this requires further validation.

Despite these recent efforts, EBV’s involvement in MS pathogenesis remains unclear, especially how it interacts with the immunogenetic factors determining susceptibility. Although we recently reported a statistical interaction between EBV antibody response and HLA-DRB1*15:01,^6,14^ the primary genetic risk for MS, how the broader genetic factors governing EBV-associated host response and IM risk relate to that of MS susceptibility is not well understood.^6,15^ This study identifies genome-wide and HLA-specific risk factors associated with high EBV antibody levels and IM history using large MS case-control cohorts from the Swedish population. We cross-evaluated these findings with recently identified genetic risk factors for MS^16^ to elucidate the complex relationship between EBV serological responses, IM and MS risk.

## Materials and methods

### Study cohorts

Samples were obtained from four Swedish MS studies^17,18^: Genes and Environment for MS (GEMS), Epidemiological Investigation of MS (EIMS), Immunomodulation and MS Epidemiology (IMSE), and STOPMS. GEMS and EIMS are nationwide case-control studies examining the genetic and environmental risk factors for prevalent and incident MS, respectively. Prevalent cases were identified from the National Swedish MS registry, while incident cases were continually enrolled through collaboration with 42 neurological clinics throughout Sweden. Controls were matched by sex, age at enrolment and region of residence in Sweden. For GEMS and EIMS, a history of IM was obtained by self-reported questionnaires with those unsure being excluded. IMSE is an ongoing phase IV clinical trial that monitors long-term safety and risk of immunomodulatory treatments for MS. Only baseline samples (i.e. before administration of monitoring treatment) were used in this study. Last, STOPMS is a local cohort enrolling new MS incident cases from the Karolinska University Hospital (Stockholm, Sweden). In cases of dual enrolment, the earliest blood sample taken closest to disease onset was used. In total, 8744 MS cases and 7229 controls were included in this study (Table 1).

**Table 1:** Summary statistics of the cohort, by multiple sclerosis status

Variable	All	MS cases	Controls
All subjects (*n*)	15 973	8744	7229
GEMS (*n*)	8906	4755	4151
EIMS (*n*)	5676	2617	3059
IMSE (*n*)	1076	1076	0
STOP-MS (*n*)	315	296	19
Age (mean ± SD)	47.5 ± 13.8	47.1 ± 14.0	48.1 ± 13.4
Sex (M:F)	4224:11 749	2445:6299	1779:5450
**EBNA-1 truncated**			
Level (mean ± SD)	5091.2 ± 1644.0	5384.9 ± 1502.4	4735.9 ± 1735.4
Status [1] (+:−) [%]	1307:2380 [64.6%]	324:1550 [82.7%]	983:830 [45.8%]
Status [2] %	86.8%	92.6%	79.8%
**EBNA-1 peptide**			
Level (mean ± SD)	807.5 ± 357.2	908.6 ± 320.8	685.2 ± 360.8
Status [1] (+:−) [%]	2083:4014 [65.8%]	554:2874 [83.8%]	1529:1140 [42.7%]
Status [2] %	60.3%	72.7%	45.4%
**VCAp18**			
Level (mean ± SD)	2608.2 ± 919.3	2702.3 ± 851.6	2494.3 ± 983.0
Status [1] (+:−) [%]	1897:2175 [53.4%]	730:1266 [63.4%]	1167:909 [43.8%]
Status [2] %	79.5%	83.6%	74.4%
**IM history**			
Status	12.5%	15.6%	9.5%

**Figure 1:**
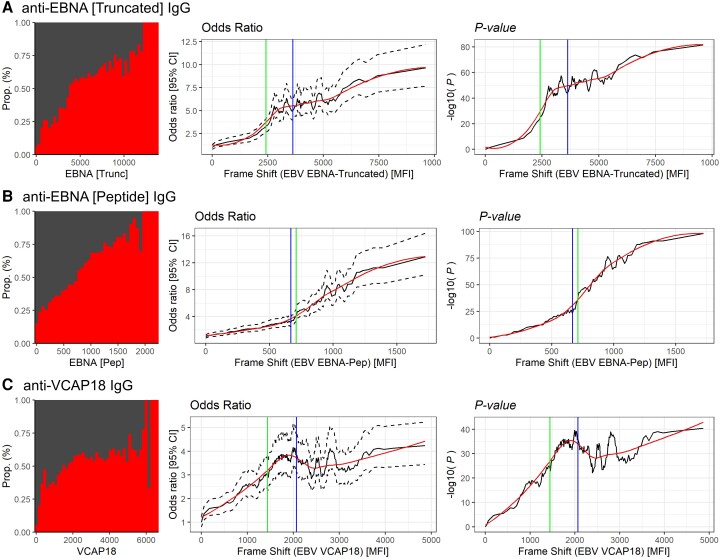
**Higher anti-EBV IgG levels associated with increasing risk for multiple sclerosis (MS).** The *left*-*side* plot illustrates the proportion of MS cases (*bottom*) compared to controls (*top*) with increasing strata of Epstein-Barr virus (EBV) antibody levels. The plots following are continuous association curves (CAC), which were determined by comparing increasing and overlapping strata (5% quantile window, +0.5% step) with the lowest 5% quantile window as reference. Line-plots for (*middle*) odds ratios along with 95% confidence intervals (CI, dotted lines) and (*right*) *P*-values are plotted for each antibody measure. The predicted effect is illustrated by the smoothed red curved. Predicted cut-off for biological effect was determined by inflection point (green line) and shown in comparison with median antibody level among controls (blue line).

Descriptive statistics include age at sampling, sex (male:female), distribution of Epstein-Barr virus (EBV) serology and infectious mononucleosis (IM) history stratified by multiple sclerosis (MS) cases and population-matched controls from four Swedish cohorts (GEMS, EIMS, IMSE, and STOP-MS). The mean (standard deviation, SD) of age at onset (AAO) and disease duration at sampling for MS cases were 33.2 (10.6) and 13.7 (12.2) years, respectively. Serology measures were IgG antibodies against the nuclear antigen EBNA-1 (truncated/peptide) and the viral capsid antigen VCAp18. ‘High/Low’ status was determined by either [1] median ± 1 SD among controls or [2] approximated by the inflection point of a continuous association curve illustrated in Fig. 1.

GEMS = Genes and Environment for MS; EIMS = Epidemiological Investigation of MS; IMSE = Immunomodulation and MS Epidemiology.

All cohorts within this study were approved by the governing Regional Ethical Review Board in Stockholm, Sweden. All study participants, including controls, were properly informed of the terms of their study and provided written consent prior to participation.

### Measuring anti-EBV IgG levels in plasma

EDTA plasma was analysed by multiplex serology for viral IgG antibodies against three EBV proteins: the 18-kDa viral capsid antigen (VCAp18) and two fragments of the Epstein-Barr nuclear antigen 1 (EBNA-1), a truncated-form [amino acids (aa) 325–641] and a smaller peptide segment (aa385–420).^19^ The latter fragment had the strongest immune response association for MS in our previous study.^6^ In summary, viral proteins were bacterially expressed as glutathione S-transferase (GST)-fusion proteins and *in situ* affinity purified directly on fluorescently distinguishable glutathione-casein (GC)-coupled bead sets.^20^ Antigen-loaded beads were combined into one bead mix and presented to primary plasma antibodies simultaneously. Formed immunocomplexes were detected using a biotinylated anti-IgG secondary antibody. This methodology allows parallel measurements of IgG antibodies against multiple antigens in one reaction. Antibodies were quantified from at least 100 beads per bead set in median fluorescence intensities (MFI) using a Luminex 200 analyser and normalized using a Box-Cox transformation.

As most of the general population have been exposed to EBV, ‘high/low’ antibody status was determined by a cut-off of ± 1 standard deviation (SD) from the median among controls.^6^ In addition, a cut-off for approximating biological effects was set using a continuous association curve (CAC), which is determined by modelling the change in risk association with MS among increasing and overlapping strata of anti-EBV IgG measures.^21^ The cut-off is then represented by the inflection point where the greatest rate of change in risk occurs (Fig. 1).

### Genotyping and HLA imputation

Individuals were genotyped using an Omniexpress Illumina chip measuring over 600 000 single nucleotide polymorphisms (SNPs). Marker quality control consisted of minor allele frequency (>2%), genotyping rate (>98%) and Hardy-Weinberg equilibrium (*P* < 0.0001). Population stratification was determined by principal component analyses (PCA) of ancestral informative markers using SmartPCA (EIGENSTRAT). Population outliers were removed along with individuals having sex discrepancy, genotype missingness (>2%), increased heterozygosity rate (*F* < mean − 3 SD) and relatedness (identity-by-descent, >0.175). In summary, a total of 6826 MS cases and 5675 controls passed quality control.

A separate custom Illumina chip (MS Replication Chip) with genotypes for ∼90 000 SNPs was utilized to impute classical HLA alleles due to its superior coverage of the HLA region.^16^ Similar marker and individual quality controls were performed before imputation, with 7062 MS cases and 6098 controls passing quality control. Classical four-digit HLA alleles were imputed using HLAIMP*02.^22^ In addition, genotypes from the MS Replication Chip were used to determine non-HLA genetic risks for MS.^16^

### Genetic risk score

Weighted genetic risk scores (wGRS) for MS were calculated using previously known HLA genetic risk alleles^23^ and the recently published 200 non-HLA risk loci.^16^ Scores were determined by the number of risk alleles carried at each locus weighed by multiplying the known effect size. A similar score was determined for each individual and EBV antibody response to compare the overall genetic effects of EBV response.

### Statistical analyses

Differences in demographic characteristics were examined with the Wilcoxon ranked-sum test or chi-square test. Odds ratios (OR) and beta-values (β) were determined by multivariable logistic and linear regression models, respectively, adjusting for age at sampling, sex and cohort type (i.e. prevalence or incidence). Joint analyses with MS cases and controls were additionally adjusted for MS affection status. All HLA allele variants with a carriage prevalence >2% were analysed in association with levels of IgG antibodies. To control for multiple comparison bias, HLA associations with *P* < 0.0005, as determined by Bonferroni correction (*n* = 100), were considered statistically significant. HLA haplotypes were determined by referencing published datasets for the European population (AlleleFrequencyNET database). Conditional HLA analyses were then performed with the top significant allele of each haplotype to estimate independent effects. General analyses, including HLA and genetic risk scores, were performed in R (version 4.2.2).

Genome-wide association (GWA) analyses were performed using an appropriate linear/logistic regression analysis in PLINK v.1.9, adjusted for age at sampling, sex, type of study and five PCA vectors. Genotype associations with a *P* < 5 × 10^−8^ were considered statistically significant, although nominal associations with significance below 10^−5^ were investigated.

## Results

### Characteristics of Epstein-Barr virus serology

The antibody measures against EBNA-1 and VCAp18 were strongly intercorrelated (*r* = 0.5–0.7, Supplementary Figs 1 and 2). Levels between the two anti-EBNA-1 measures were expectedly disproportionate, with more having antibody response against the longer and more inclusive truncated protein likely due to additional antibody binding sites outside the smaller peptide fragment. The relative proportion of antibody levels (i.e. peptide/truncated) for each individual was determined by transforming the pair over the axis of best linear fit, as shown in Supplementary Fig. 1. The resulting index measure (‘pepIndex’) was only correlated with the antibody levels against the smaller peptide fragment (*r* = 0.69) but not the truncated protein (*r* < 0.01).

Among controls, females had a slightly higher anti-EBNA-1 truncated IgG level than males (Supplementary Table 1). Age at sampling was associated with a general increase in antibody levels. Longer disease duration was primarily associated with higher anti-VCAp18 IgG and, to a lesser extent, with anti-EBNA-1 truncated but not with anti-EBNA-1 peptide (Supplementary Fig. 3).

### Epstein-Barr virus antibody levels and infectious mononucleosis are associated with multiple sclerosis

As expected, high EBV antibody levels and IM history were associated with a higher risk for MS and, in line with our previous study,^6^ antibodies against EBNA-1 peptide had the strongest risk association with MS (Fig. 1, seropositivity shown in Supplementary Fig. 2). Importantly, this observed MS risk was not stepwise but increased gradually with higher antibody levels. The risk association was notably higher for the anti-EBNA-1 peptide, with the relative MS risk among the highest five percentile antibody response being ∼12× higher than the lowest five percentile.

The overall association between anti-EBNA-1 IgG and MS decreased with increasing age (Supplementary Fig. 4), likely due to the general increase in EBV antibody levels with age among the controls (Supplementary Table 1). On the contrary, MS association with anti-VCAp18 increased slightly with age, while all antibody associations with MS dropped notably after age 50. The reported history of IM did not show any effect modification by age (Supplementary Fig. 5). Males showed a slightly stronger risk association for all anti-EBV IgG measures with MS, although females generally had a higher antibody response (Supplementary Table 2).

History of IM was associated with an increased anti-EBNA-1 peptide IgG among both cases and controls (*P* = 1.8 × 10^−4^) but was not associated with EBNA-1 truncated or VCAp18 (Supplementary Table 3). This effect was further supported by the stronger association with pepIndex (*P* = 6.26 × 10^−5^), suggesting a specific relationship between IM and the response against the smaller peptide fragment of EBNA-1. However, IM history did not affect the associated risk between anti-EBV IgG responses and MS (Supplementary Table 4).

### HLA variants, including DRB1*15:01, are associated with anti-Epstein-Barr virus IgG levels

**Figure 2:**
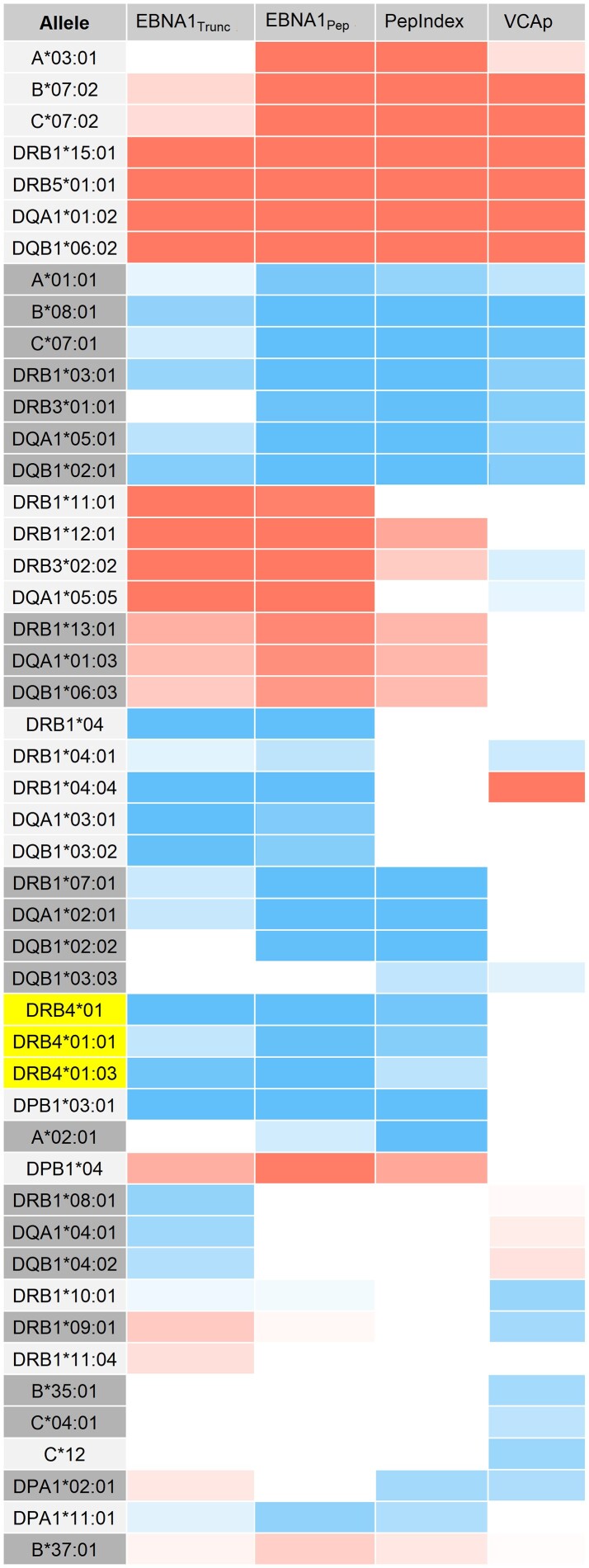
**HLA haplotypes associated with anti-EBV IgG levels.** The figure summarizes all HLA alleles associated to anti-EBV IgG levels with significance of *P* < 10^–4^, organized by corresponding haplotype. Heat map gradients were used to illustrate both significance level (darker = more significant) and type of effect: risk (red) and protective (blue). HLA-DRB4*01 (yellow) and its subtypes 01:01 and 01:03 are associated with the two haplotypes, DRB1*04:01 and DRB1*07:01. Values showing the beta (β) and significance (*P*) among all subjects adjusted for sex, age at sampling, multiple sclerosis affection status and six principal components analysis (PCA) vectors are provided in the Supplementary material.

Several HLA haplotypes were independently associated with anti-EBV IgG levels, as summarized in Fig. 2 (see also Supplementary Table 5). The haplotypes of DRB1*15:01 and DRB1*03:01 were the primary genetic associations with EBV antibody measures and, notably, are also the primary genetic associations for MS. These associations were present for all antibody measures among MS cases and controls. Additionally, DRB1*04:01 also showed an association with all three antigens but was more significantly associated with anti-VCAp18 IgG levels. However, the remainder of the common haplotype of DRB1*04:01 was not associated, suggesting different haplotypes.

Although many overlapping HLA associations existed between the different antibody measures, some associations seem specific to one measure (Fig. 2). For example, haplotypes of DRB1*11/12:01 and 13:01 were only positively associated with anti-EBNA-1 IgG levels and not VCAp18. Furthermore, analyses against the pepIndex suggest this genetic association likely targets the truncated measure. In contrast, A*02:01 was only associated with EBNA-1 peptide and only among cases (β = −43.8, *P* = 8.52 × 10^−9^) and not controls (β = −3.15, *P* = 0.734). Last, carriage of A*02:01 was the only genetic association for the history of IM (OR = 0.78, *P* < 5 × 10^−5^).

All serological and IM history associations with MS remained after adjusting for potential HLA genetic confounding (Table 2).

**Table 2:** Multivariable regression analyses examining the MS risk association for serological response against EBV and IM history

	Unadjusted			HLA adjusted^a^		
	β/OR	SE/(CI)	*P*	β/OR	SE/(CI)	*P*
**EBNA-1 truncated**						
Level	604	25.58	3.60 × 10^−121^	528	29.05	6.60 × 10^−73^
Status [1]	5.43	(4.65, 6.35)	2.10 × 10^−100^	5.16	(4.27, 6.24)	9.60 × 10^−65^
Status [2]	3.08	(2.79, 3.41)	1.40 × 10^−108^	2.90	(2.58, 3.27)	1.80 × 10^−68^
**EBNA-1 peptide**						
Level	218	5.43	<1.00 × 10^−250^	192	6.2	1.50 × 10^−202^
Status [1]	6.84	(6.07, 7.71)	2.90 × 10^−216^	5.74	(4.97, 6.63)	2.70 × 10^−125^
Status [2]	3.13	(2.93, 3.35)	4.30 × 10^−248^	2.81	(2.60, 3.04)	2.90 × 10^−149^
**PepIndex [peptide/truncated]**						
Level	233	6.74	<1.00 × 10^−250^	208	7.76	4.90 × 10^−154^
Status [1]	4.76	(4.24, 5.35)	2.40 × 10^−150^	4.07	(3.55, 4.67)	3.80 × 10^−89^
Status [2]	2.96	(2.75, 3.18)	3.90 × 10^−184^	2.72	(2.50, 2.96)	1.20 × 10^−118^
**VCAp18**						
Level	193	14.24	1.90 × 10^−41^	170	15.7	4.60 × 10^−27^
Status [1]	2.14	(1.87, 2.45)	1.40 × 10^−27^	2.03	(1.74, 2.37)	4.20 × 10^−19^
Status [2]	1.74	(1.60, 1.88)	4.50 × 10^−42^	1.67	(1.53, 1.83)	2.00 × 10^−28^
**IM history**						
Status	1.87	(1.67, 2.10)	4.70 × 10^−27^	1.78	(1.56, 2.02)	6.80 × 10^−19^

Summary of linear and logistic regression analyses assessing the association with multiple sclerosis (MS) affection status for anti-EBV IgG levels or high/low status, along with the derived index of EBNA-1 peptide and truncated antibody levels (PepIndex) and self-reported infectious mononucleosis (IM) history. High/low status was determined by either [1] median ± 1 SD (standard deviation) among controls or [2] approximated by the inflection point of a continuous association curve illustrated in Fig. 1. Values are either beta (β), standard error (SE) and significance (*P*); or odds ratio (OR), 95% confidence interval (CI) and significance. EBV = Epstein-Barr virus. EBV = Epstein-Barr virus.

^a^Models were adjusted for sex, age at sampling, and study type along with associated HLA allele variants for each measure.

### Genome-wide association analysis of Epstein-Barr virus antibody levels

There were no significant genetic associations other than those in the HLA region for any EBV antibody measures (Supplementary Figs 6 and 7). Conditioning on the previously identified HLA haplotypes (Supplementary Table 5) mostly depleted the association within chromosome six, indicating that the primary genetic associations are likely variants from within the HLA regions (Supplementary Fig. 8 and Supplementary Table 6). Several nominal associations were discovered outside the HLA region, as listed in Supplementary Table 7. SNP rs9313900, located in the intron of the *SGCD* gene, was associated with high/low status and antibody levels against EBNA-1 peptide (*P* = 3.67 × 10^−7^). Similarly, anti-VCAp18 serostatus showed nominal association peaks with rs2304669 (*P* = 6 × 10^−7^) and rs2304673 (*P* = 7 × 10^−6^), which are located within the genes *PER2* (chr2.q37.3) and *TRAF3IP1* (chr2.q37.3), respectively. Only one of the four previously reported genetic risk loci for anti-EBNA-1 IgG titres, rs2255214, was replicated (*P* < 0.05) (Supplementary Table 8).^12^

### Genetic risks for high anti-EBNA-1 IgG levels are associated with multiple sclerosis

**Figure 3:**
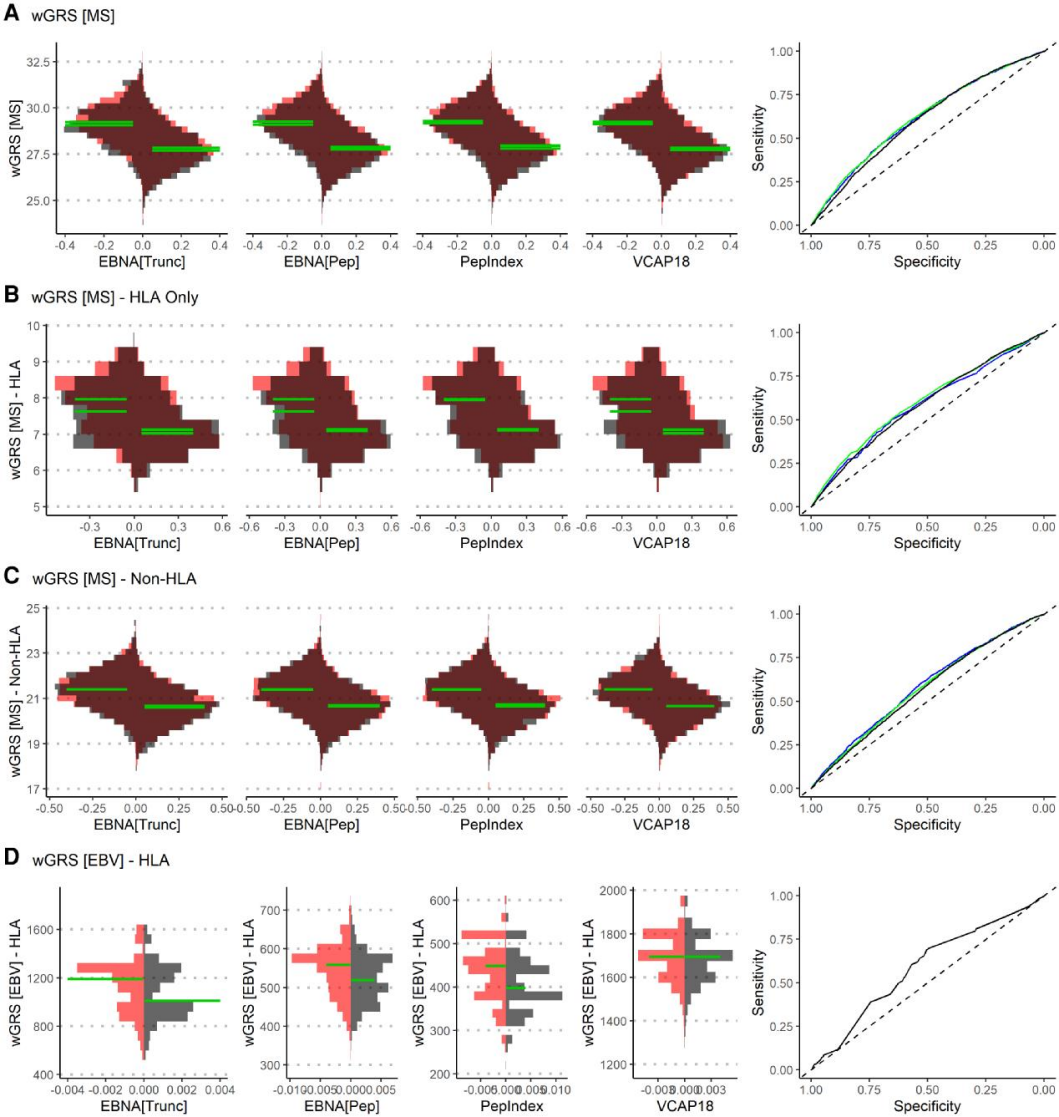
**Cross-comparing the distribution of weighted genetic risk scores (wGRS) for MS and anti-EBV IgG levels.** Asymmetric bean plots illustrate the distribution of weighted risk scores for either (i) multiple sclerosis (MS) between high/low antibody response [continuous association curves (CAC) cut-off, **A**–**C**]; or (ii) anti-EBV IgG levels between MS cases and controls (**D**). See Supplementary Tables 9–11 for additional details. Analyses of weighted genetic risk scores (wGRS) for MS (**A**) were also conducted separately for (**B**) HLA and (**C**) non-HLA genetic risks. For **A**–**C**, *left*- and *right*-side distributions represent low and high antibody response, respectively, based on the inflection-based method. Red and grey distributions correspond to MS cases and controls, respectively. The median is displayed for each distribution and a receiver operating characteristic (ROC) curve of each comparison is illustrated on the *right*.

The distribution of wGRS for MS, including both HLA and non-HLA, and the HLA GRS for each of the EBV serology measures are shown in Fig. 3. The overall genetic risk for high anti-EBNA-1 truncated and peptide was higher among MS cases than controls (Fig. 3D). However, the genetic risk for anti-VCAp18 IgG was not as strongly associated with MS as anti-EBNA-1. Overall, these results suggest a bidirectional relationship, with onset of MS also leading to increased antibodies against EBV. However, the distribution plots indicate that wGRS for anti-EBNA-1 IgG is the primary association with MS. Further examination of the various EBNA-1 fragments examined in our previous study^6^ showed aa385–420 as the only fragment associated with HLA and non-HLA MS wGRS among MS cases and controls (Supplementary Fig. 9).

## Discussion

Our findings indicate that higher IgG antibody levels against EBV, particularly the aa385–420 peptide fragment of EBNA-1, were associated with increasingly higher risk for MS,^12^ up to 12× the reference risk. However, increased antibody levels against certain EBV antigens may also develop as a consequence of disease pathology following onset. This is supported by the positive correlation between anti-VCAp18 IgG levels and disease duration among MS patients, which was not observed for anti-EBNA-1, and likely explains why the association between anti-EBNA-1 IgG and MS is predominantly displayed among those sampled at younger ages while the reverse pattern was observed with VCAp18.

A potential explanation for the increased EBV antibodies could be T-cell exhaustion resulting in decreased EBV-specific CD4/CD8+ T cells^24,25^ and poorly checked immune control of EBV related to less efficient presentation of EBV-related antigens. This may facilitate the proliferation of EBV, which is further evidenced by the reported increase in EBV viral load.^26,27^ As VCA is expressed during viral replication, the higher anti-VCAp18 IgG levels with longer disease duration among MS persons also reflect a higher degree of virus activation/replication during MS. Interestingly, one study has found a relation between anti-VCA antibodies and grey matter pathology.^28^

However, the mechanism of EBV infection in MS aetiology, particularly concerning EBNA-1, remains unclear. Although one could consider several ways in which EBV could, in part, cause or propagate the disease, we consider two appropriate options here: (i) defective immune control of EBV, which in turn, by unknown mechanisms, drives MS, perhaps by a direct attack against EBV present in the brain^29^; or (ii) a case of molecular mimicry, where viral antigens mimic self CNS proteins with ensuing pathogenic autoimmunity.^30-33^

HLA variants are the main host genetic factors influencing EBV antibody response and the primary genetic susceptibility for MS risk. There are several overlapping HLA variants, including DRB1*08:01, DQB1*03:01 and particularly the DRB1*15:01 and DRB1*03:01 haplotypes, which are not only the primary genetic associations for EBV serology but also for MS.^6,14,23^ Interestingly, although both alleles DRB1*15:01 and DRB1*03:01 are associated with higher MS risk, DRB1*03:01, unlike DRB1*15:01, is associated with lower EBV antibody levels, possibly indicating different mechanisms of association. DRB1*15:01 was also similarly associated with EBV viral load in a previous study, although it was not directly correlated with IgG levels against either EBNA-1 or viral capsid antigen.^26^ This is consistent with HLA-DRB1*15:01 being a poor class II antigen in the immune defence against EBV, as shown experimentally in humanized mice.^34^ Class II antigens also serve as a (gp42)-viral receptor facilitating EBV entry into B lymphocytes; therefore, sequence variability may affect the binding affinity of the virus.^35^ However, considering the role of HLA in antigen presentation, overlapping associations are likely an indication of molecular mimicry and the cross-reactivity between EBV and auto-antigens.

Several CNS host antigens mimic EBNA-1 epitopes, displaying conspicuous immune reactivities in persons with MS compared to controls. One of the potential candidates is alpha β-crystallin (aβ-crystallin), a highly specific heat-shock protein with sequence homology to EBNA-1.^36^ EBV-infected lymphocytes have been shown to express aβ-crystallin, potentially leading to increased antigen accumulation in local inflammatory lesions of MS.^37^ We recently showed that an amino acid sequence homology between the EBV aa399–415 and aβ-crystallin aa8–27 displayed increased T and B cell responses in MS.^31^ Myelin basic protein (MBP), important in maintaining myelin structure, has been a popular target of immuno-reactivity experiments, and studies examining MBP-specific T-cell clones from MS patients have shown cross-reactivity with EBV-encoded DNA polymerase.^38,39^ In addition, sera from persons with MS recognizing an EBNA-1 epitope also recognized an MBP epitope: aa205–224 without amino acid sequence homology. Recently, anoctamin-2 has been proposed as an autoimmune target in MS with antibody response associated with higher MS risk, along with cross-reactivity to specific peptides of EBNA-1.^30^ Finally, the sequence homology between EBNA-1 and GlialCAM has shown potent immune reactivity in MS.^32^ While the steady increase in MS risk related to EBNA-1 antibody levels is consistent with a direct pathological role of that immunity with the multitude of potential mimicry epitopes, the degree and extent of molecular mimicry *in vivo* for MS pathogenesis remain to be firmly defined.

In contrast to serology, IM incidence was primarily associated with A*02:01, a major protective allele for MS. Several studies have also reported A*02:01 to be protective against EBV-related Hodgkin’s lymphoma,^40-42^ likely from a stronger LMP2A (latency II)-specific CD8+ T cell response.^43^ This may explain the differences in genetic and MS risk associations among IM history and EBNA-1 (latency I–III) and VCAp18 (lytic) serology. Another possible explanation stems from A*02 capability of regulating the type 1 interferon system, which may affect the severity of EBV infection and subsequent symptomatic presentation as IM, disconnecting that phenomenon from antigen presentation to T cells.^44^ Their independent association with MS supports the genetic dissimilarity between EBV serology and IM history. However, IM history and A*02:01 were associated with antibody response against the peptide fragment of EBNA-1, suggesting their relationship may be influenced by the timing of MS onset and EBV infection.

There were few genetic associations observed outside of the HLA region. One nominal candidate may suggest the involvement of *EGF* (rs2220427). Previous studies have shown *EGF* facilitates the cellular entry of EBV when infecting epithelial cells, which may increase the overall risk of EBV infection while also regulating viral activity and infected cell growth.^45^ Of the non-HLA genetic risks for MS, only rs2255214, near the *CD86* gene, was significantly associated with EBV serology and was the only genetic association replicated from previous genetic EBV studies. *CD86* is an important co-stimulatory protein for T cell activation with increased expression in monocytes of MS patients and responsive to interferon treatment.^46^ Studies have shown that EBNA-2 may regulate several MS susceptibility loci, including the *CD86* gene region affecting expression,^3,25,47^ perhaps modifying T cell activity and MS susceptibility. The potential mechanisms of other genetic risk loci are highly speculative, either playing a role in the direct immune response modulating EBV activity or possibly the susceptibility and timing of initial EBV infection. However, these findings will require further investigations to validate and establish potential mechanisms of action.

Similar to previous genetic studies,^13^ we could only identify a handful of associations within the HLA region, limiting the quality of genetic instruments and the capabilities for examining causal inference. This was also a limitation for investigating the role of IM, which will require a larger cohort to determine adequate genetic associations. Furthermore, HLA is highly pleiotropic, influencing many immunological processes and associated with numerous inflammatory diseases and pathogenic factors. Although previous findings have indicated a possible causal relationship between EBV and MS,^6,12^ further studies are necessary to investigate how these genetic components influence the relationship between EBV and MS, especially identifying better genetic instruments for assessing the risk of EBV infection and host immune response.

In summary, our findings provide an important step for understanding the role of EBV in MS pathology, emphasizing the importance of EBNA-1 peptide aa(385–420) and, in particular, the gradual MS risk increase in relation to higher EBNA-1 antibody levels. Further investigation will be required to disseminate the serological role for other proteins, notably EBNA-2 and LMP-1, along with relations to viral load, which has overlapping associations but limited correlation to serological response.^26,27^ Although EBV is the most prominent viral risk factor, other infectious agents, particularly other herpes viruses, such as cytomegalovirus and human herpes virus 6, are known to modify MS risk and disease activity.^4^ Further studies should examine the causal inference of these viruses and their potential interaction with EBV and HLA in MS pathogenesis.

## Supplementary Material

awae110_Supplementary_Data

## Data Availability

The anonymized raw data used to support this study will be made available by the authors to any qualified researcher upon request.
