# Supplementary material for: Genetics of immune response to Epstein-Barr virus: prospects for multiple sclerosis pathogenesis
Source: Brain. 2024 Apr 17;147(10):3573–82. doi: 10.1093/brain/awae110 (PMC11449136; doi:10.1093/brain/awae110)
Supplement: awae110_Supplementary_Data [file awae110_supplementary_data.pdf]

SUPPLEMENTARY TABLES & FIGURES

**Figure S1.** Correlation between anti-EBV IgG measures.	2
**Figure S2.** Distribution and seropositivity rate of anti-EBV IgG measures.	3
**Table S1.** Distribution of anti-EBNA-1 truncated IgG levels stratified by age group, sex, IM history, and MS status.	4
**Figure S3.** Correlation between disease duration and anti-EBV IgG levels among MS cases.	5
**Figure S4.** Effect modification of the risk association between anti-EBV IgG levels and MS status by age at sampling.	6
**Figure S5.** Effect modification of the risk association between anti-EBV IgG levels and MS status by age at sampling and IM history.	7
**Table S2.** Association between MS and both anti-EBV IgG level and status, stratified by sex.	8
**Table S3.** Association between IM history and anti-EBV IgG levels, stratified by MS status.	9
**Table S4.** Association between MS and both anti-EBV IgG level and status, stratified by IM history.	10
**Table S5a.** Summary of linear regression analyses examining the association between HLA variants and anti-EBNA-1 truncated IgG levels.	11
**Table S5b.** Summary of linear regression analyses examining the association between HLA variants and anti-EBNA-1 peptide IgG levels.	13
**Table S5c.** Summary of linear regression analyses examining the association between HLA variants and PepIndex.	16
**Table S5d.** Summary of linear regression analyses examining the association between HLA variants and anti-VCAp18 IgG levels.	18
**Figure S6.** Genome-wide associations to anti-EBV IgG levels and IM history.	20
**Figure S7.** Genome-wide associations to anti-EBV IgG levels and IM history, stratified by MS affection status.	21
**Figure S8.** Genome-wide associations to anti-EBV IgG levels conditioning for associated HLA allele variants.	22
**Table S6.** Residual HLA genetic associations to anti-EBV IgG levels below significance of P < 5x10-8.	23
**Table S7.** Primary non-HLA genetic associations to anti-EBV IgG levels below nominal significance of $P < 10^{-5}$.	24
**Table S8.** Replication of previously established genetic associations to anti-EBNA-1 serology.	26
**Table S9.** Weights used for calculating anti-EBV IgG polygenetic risk score.	27
**Table S10a.** Association between anti-EBV IgG levels and the individual HLA MS genetic risks.	28
**Table S10b.** Association between anti-EBV IgG levels and the individual non-HLA MS genetic risks.	29
**Table S11.** Bi-directions genetic risk score association for anti-EBV IgG levels and MS.	33
**Figure S9.** Association between genetic risk for MS and antibody levels against different EBNA-1 peptide fragments.	34

Figure S1. Correlation between anti-EBV IgG measures.

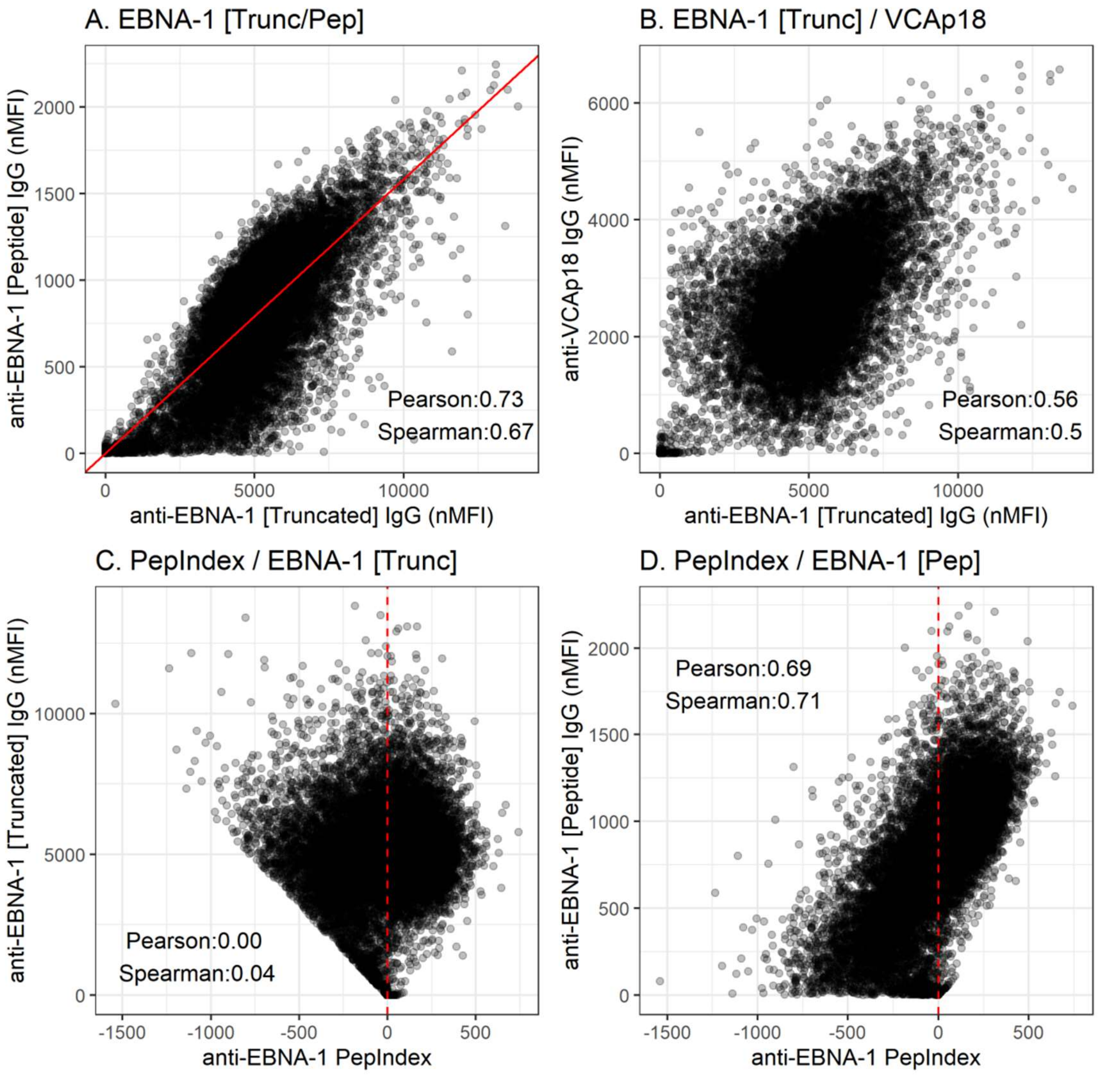

Scatterplots illustrate the correlation between IgG levels of anti-EBNA-1 truncated and either [A] anti-EBNA-1 peptide or [B] anti-VCAp18. Pearson and Spearman correlation coefficients are listed for each plot. The transformation axis (red line) in panel A illustrates the base-reference (i.e., zero) used for determining the EBNA1 index measure (pepIndex), which is the proportion in IgG antibody levels against EBNA-1 peptide and truncated. The resulting index measures is strongly correlated with anti-EBNA1 peptide [D] but not truncated IgG measures [C] which assists in comparing associations between the two correlated EBNA-1 measures.

Figure S2. Distribution and seropositivity rate of anti-EBV IgG measures.

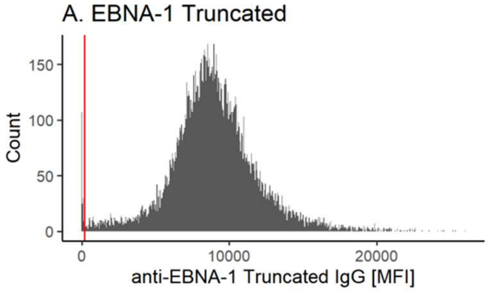

Anti-EBNA-1 [Trunc] Seropositive (+)

	VCAp18 (-)	VCAp18 (+)
EBNA-1 Pep (-)	36	333
EBNA-1 Pep (+)	17	15415

Anti-EBNA-1 [Trunc] Seronegative (-)

	VCAp18 (-)	VCAp18 (+)
EBNA-1 Pep (-)	155	13
EBNA-1 Pep (+)	3	1

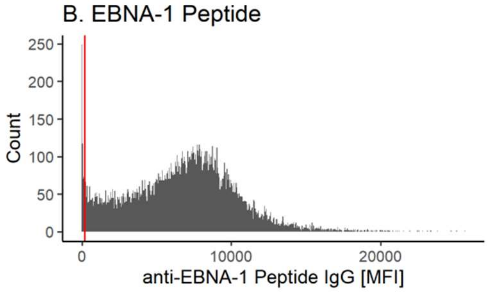

Anti-EBNA-1 [Pep] Seropositive (+)

	VCAp18 (-)	VCAp18 (+)
EBNA-1 Trunc (-)	3	1
EBNA-1 Trunc (+)	17	15415

Anti-EBNA-1 [Pep] Seronegative (-)

	VCAp18 (-)	VCAp18 (+)
EBNA-1 Trunc (-)	155	14
EBNA-1 Trunc (+)	36	332

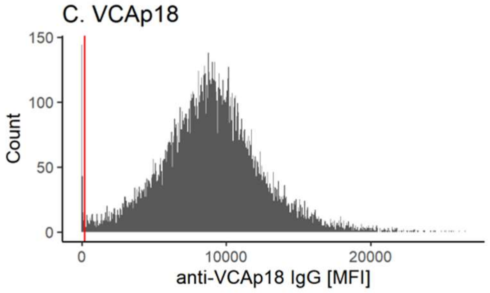

Anti-VCAp18 Seropositive (+)

	EBNA-1 Trunc (-)	EBNA-1 Trunc (+)
EBNA-1 Pep (-)	14	332
EBNA-1 Pep (+)	1	15415

Anti-VCAp18 Seronegative (-)

	EBNA-1 Trunc (-)	EBNA-1 Trunc (+)
EBNA-1 Pep (-)	155	36
EBNA-1 Pep (+)	3	17

Histograms illustrate the distribution of anti-EBV IgG levels including [A] EBNA-1 truncated, [B] EBNA-1 peptide, and [C] VCAp18. Serostatus was determined by the low-distribution peak defined by a cutoff of MFI < 200 (red line). The overlapping frequencies between different antibody measures are shown in the right-hand tables. Seropositivities for EBNA-1 truncated, EBNA-1 peptide, and VCAp18 were 99.90%, 98.95%, 99.87% for MS cases and 97.75%, 93.84%, and 97.23% for matched controls, respectively. Differences in seropositivity between cases and controls were significant for all measures, $P < 10^{-38}$.

Table S1. Distribution of anti-EBNA-1 truncated IgG levels stratified by age group, sex, IM history, and MS status.

	Sex			IM History		
**Age**	**N(M:F)**	**Male**	**Female**	**N(+/-)**	**IM +**	**IM -**
**MS Cases**						
<20	42:71	9767 ± 4.1	9851 ± 3.7	12:46	11118 ± 4.4	9108 ± 2.3
≥20-30	256:716	9093 ± 2.9	9252 ± 2.7	124:415	8866 ± 2.3	9066 ± 2.4
≥30-40	530:1306	9553 ± 2.8	9222 ± 2.7	244:804	9268 ± 2.5	9250 ± 2.6
≥40-50	573:1550	9707 ± 2.9	9531 ± 2.8	253:1063	9577 ± 2.7	9425 ± 2.6
≥50-60	523:1390	9734 ± 3.0	9524 ± 2.8	170:1203	9407 ± 2.5	9563 ± 2.7
≥60	521:1266	9414 ± 2.8	9447 ± 3	93:1307	9594 ± 3.1	9428 ± 2.9
**Controls**						
<20	18:36	4098 ± 4.2	7175 ± 4.2	4:47	7597 ± 0.7	6257 ± 4.6
≥20-30	139:480	7364 ± 3.6	7556 ± 3.2	92:459	7567 ± 2.6	7449 ± 3.4
≥30-40	360:1121	7779 ± 3.5	7950 ± 3.1	159:1118	8301 ± 3.1	7868 ± 3.2
≥40-50	385:1454	7983 ± 3.3	8325 ± 3.1	176:1357	8367 ± 3.0	8213 ± 3.1
≥50-60	425:1276	8349 ± 3.1	8477 ± 3.1	93:1279	8094 ± 2.8	8495 ± 3.1
≥60	452:1083	9072 ± 3.2	8696 ± 3.1	49:1210	9392 ± 2.6	8777 ± 3.2

Mean and standard deviation (SD) of anti-EBNA-1 IgG levels are listed stratified by age group, sex (M=male; F=female), IM history ("+"=yes; "-"=no), and MS affection status. SD are scaled 1:1000. Anti-EBNA-1 truncated IgG levels increases with age but the difference in antibody levels between MS cases and controls become smaller with increasing age. Stratification by either sex and IM history did not affect change in anti-EBNA1 truncated IgG levels with age.

Figure S3. Correlation between disease duration and anti-EBV IgG levels among MS cases.

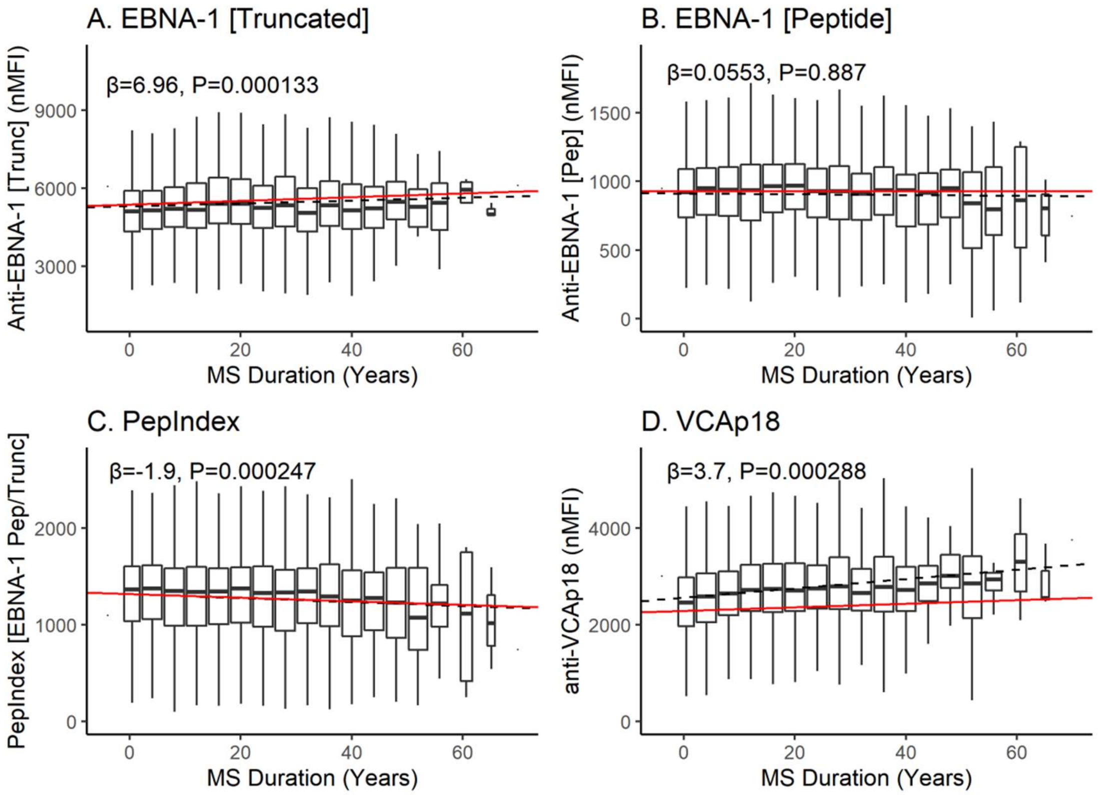

Boxplots illustrate the changes in the distribution of anti-EBV IgG levels with increasing duration of disease from onset among MS cases. A linear regression model was fitted both with (red) and without (black) adjusting for age at sampling. Both the beta coefficient (β) and significance (P) of each age-adjusted model are listed. Antibody levels against EBNA-1 truncated and particularly VCAp18 increased drastically with the duration of disease; however, antibody levels against EBNA-1 peptide remained stable.

Figure S4. Effect modification of the risk association between anti-EBV IgG levels and MS status by age at sampling.

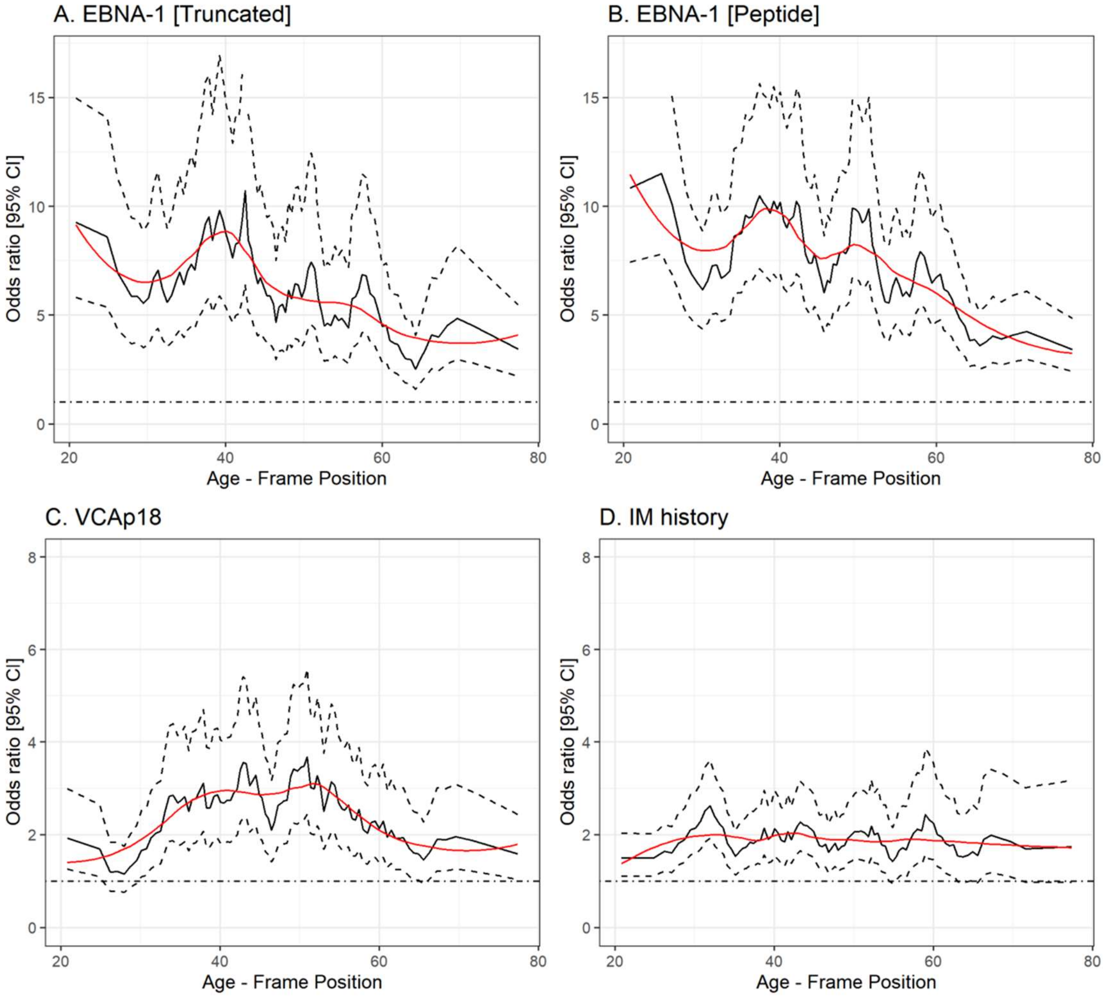

Plots illustrate the association between either [A-C] high/low antibody response or [D] IM history and risk for MS development, stratified continuously by age at sampling. Antibody measures consist of IgG antibodies against [A] EBNA-1 truncated, [B] EBNA-1 peptide, and [C] VCAp18. High/low antibody response was determined by median ± 1 SD among controls although similar results were seen using the inflection point cutoff illustrated in Figure 1. Line plots are continuous association curves determined by examining the association within increasing and overlapping age strata (increment=1%, window size=10%). A multivariable logistic regression model adjusting for sex and study type was performed for each stratum. The odds ratio (OR; black solid line) and 95% confidence intervals (95% CI; black dotted line) along with a smoothed curve (red line) approximating the predicted effect are plotted above.

Figure S5. Effect modification of the risk association between anti-EBV IgG levels and MS status by age at sampling and IM history.

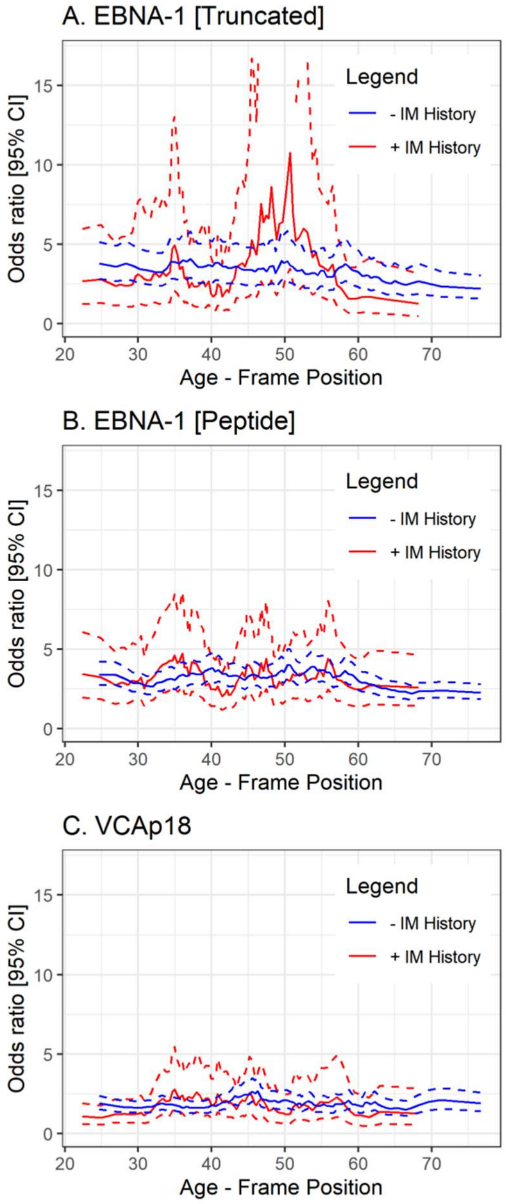

Similar to Figure S3, plots illustrate the association between either [A-C] high/low antibody response and risk for MS development stratified dichotomously by IM history and continuously by age at sampling. Antibody measures consist of IgG antibodies against [A] EBNA-1 truncated, [B] EBNA-1 peptide, and [C] VCAp18. Due to limitations in sample size as a result of IM history stratification, analyses used high/low antibody response as determined by inflection point (Figure 1). History of IM was determined by self-reported questionnaire ("+"/previous IM history, n=1469; "-"/no IM history, n=10308). Line plots are continuous association curves determined by examining the association within increasing and overlapping age strata (increment=1%, window size=15%). A multivariable logistic regression model adjusting for sex and study type was performed for each stratum. The odds ratio (OR; black solid line) and 95% confidence intervals (95% CI; black dotted line) along with a smoothed curve (red line) approximating the predicted effect are plotted above.

Table S2. Association between MS and both anti-EBV IgG level and status, stratified by sex.

	Female (n=11749)			Male (n=4224)		
	β/OR	SE/(CI)	P	β/OR	SE/(CI)	P
**EBNA-1 Truncated**						
Level	577	29.3	4.50E-85	681	52.21	3.60E-38
Status [1]	5.24	(4.37,6.29)	3.10E-71	6.12	(4.53,8.33)	2.70E-31
Status [2]	3.07	(2.74,3.45)	1.50E-79	3.12	(2.57,3.78)	6.60E-31
**EBNA-1 Peptide**						
Level	212	6.27	3.90E-239	237	10.9	1.50E-99
Status [1]	6.63	(5.77,7.64)	4.70E-153	7.47	(5.94,9.43)	2.40E-65
Status [2]	3.10	(2.87,3.35)	1.30E-181	3.22	(2.83,3.67)	1.80E-68
**PepIndex [Peptide / Truncated]**						
Level	228	7.85	7.10E-180	244	13.19	8.30E-74
Status [1]	4.62	(4.03,5.29)	5.90E-109	5.22	(4.13,6.61)	2.90E-43
Status [2]	2.86	(2.63,3.11)	2.30E-131	3.29	(2.84,3.82)	5.30E-55
**VCAp18**						
Level	165	16.32	5.40E-24	275	28.98	4.30E-21
Status [1]	2.03	(1.73,2.38)	4.60E-18	2.54	(1.95,3.32)	8.00E-12
Status [2]	1.67	(1.52,1.83)	2.00E-26	1.96	(1.69,2.28)	1.00E-18
**IM History**						
Status	1.82	(1.6,2.07)	6.60E-20	2.09	(1.63,2.69)	6.30E-09

Summary of linear and logistic regression analyses analyzing association between MS and either anti-EBV IgG levels or serostatus, respectively. Analyses were stratified by gender (female=11749, male=4224). Models were adjusted for age at sampling and study type. High/low status was determined by either [1] median +/- one standard deviation (SD) among controls or [2] approximated by the inflection point of a continuous association curve illustrated in Figure 1. Values are either beta (β), standard error (SE), and significance (P); or odds ratio (OR), 95% confidence interval (CI), and significance.

Table S3. Association between IM history and anti-EBV IgG levels, stratified by MS status.

	MS Case (n=5734)			Control (n=6043)			All Subjects		
**Measure**	**β**	**SE**	**P**	**β**	**SE**	**P**	**β**	**SE**	**P**
EBNA-1 Trunc	16.0	52.2	0.759	111.3	75.1	0.138	65.5	44.4	0.140
EBNA-1 Pep	31.5	11.5	0.00621	38.0	15.9	0.0168	35.8	9.60	1.80E-04
PepIndex	53.4	15.6	0.00064	37.9	18.1	0.0362	47.2	11.8	6.26E-05
VCAp18	-31.6	30.0	0.293	78.7	41.8	0.0596	17.4	25.0	0.487

Summary of linear regression analyses examining the association between anti-EBV IgG levels and self-reported IM history. Only antibodies against EBNA-1 peptide were increased with IM history and this discrepancy in association between the truncated and peptide fragment is further evidenced by the strong association to PepIndex.

Table S4. Association between MS and both anti-EBV IgG level and status, stratified by history of IM.

	+ IM History (n=1469)			- IM History (n=10308)		
	**β/OR**	**SE/(CI)**	**P**	**β/OR**	**SE/(CI)**	**P**
**EBNA-1 Truncated**						
Level	564	78.61	1.20E-12	574	31.41	2.40E-73
Status [1]	7.01	(3.94,12.88)	9.80E-11	5.4	(4.42,6.62)	4.70E-60
Status [2]	2.81	(2.02,3.93)	9.30E-10	3.18	(2.79,3.62)	3.80E-69
**EBNA-1 Peptide**						
Level	214	17.15	4.80E-34	211	6.74	1.70E-205
Status [1]	9.5	(6.08,15.16)	3.30E-22	6.51	(5.6,7.59)	7.50E-130
Status [2]	3.12	(2.5,3.91)	2.10E-23	3.09	(2.85,3.36)	1.60E-157
**PepIndex [Peptide / Truncated]**						
Level	237	22	5.10E-26	227	8.27	1.10E-159
Status [1]	4.93	(3.27,7.52)	6.00E-14	4.74	(4.10,5.49)	3.30E-97
Status [2]	3.15	(2.48,4.03)	3.00E-20	2.84	(2.59,3.10)	7.90E-115
**VCAp18**						
Level	115	44.01	0.009	197	17.71	1.70E-28
Status [1]	1.84	(1.14,3.00)	0.013	2.15	(1.81,2.56)	3.80E-18
Status [2]	1.56	(1.20,2.01)	0.00074	1.83	(1.65,2.02)	9.80E-32

Summary of linear and logistic regression analyses analyzing association between MS and either anti-EBV IgG levels or serostatus, respectively. Analyses were stratified by self-reported history of IM (IM[yes]=1469, IM[no]=10308). Models were adjusted for sex, age at sampling, and study type. High/low status was determined by either [1] median +/- one standard deviation (SD) among controls or [2] approximated by the inflection point of a continuous association curve illustrated in Figure 1. Values are either beta-coefficient (β), standard error (SE), and significance (P); or odds ratio (OR), 95% confidence interval (CI), and significance.

Table S5a. Summary of linear regression analyses examining the association between HLA variants and anti-EBNA-1 truncated IgG levels.

	MS Cases (n=7062)				Controls (n=6098)				All Subjects					
**Allele**	**β**	**P**	**β***	**P***	**β**	**P**	**β***	**P***	**β**	**SE**	**P**	**β***	**SE***	**P***
*Haplotype: A*03:01-B*07:02-C*07:02-DRB1*15:01-DRB5*01:01-DQA1*01:02-DQB1*06:02*														
A*03:01	49.5	0.172	32.9	0.368	19.8	0.68	10.9	0.82	37.1	29.3	0.206	25	29.5	0.396
B*07:02	32.4	0.369	15.6	0.674	152	0.00185	155	0.0016	79.4	29.5	0.0071	73.8	30	0.0139
C*07:02	22.5	0.531	13.2	0.719	153	0.00143	158	0.0011	75.6	29.2	0.00961	76	29.6	0.0103
DRB1*15:01	249	3.60E-12	249	9.10E-11	279	7.12E-09	322	8.43E-11	262	29.1	2.48E-19	283	30.7	2.96E-20
DRB5*01:01	250	2.84E-12	250	8.34E-11	285	3.05E-09	329	3.01E-11	266	29.1	7.19E-20	287	30.6	9.24E-21
DQA1*01:02	243	3.61E-11	243	9.00E-10	237	1.18E-07	290	5.06E-10	242	28.6	3.36E-17	269	30.4	8.76E-19
**DQB1*06:02**	264	1.38E-13	265	4.33E-12	272	1.91E-08	314	2.73E-10	269	29.2	3.45E-20	290	30.6	3.60E-21
*Haplotype: DRB1*11:01/12:01-DRB3*02:02-DQA1*05:05*														
DRB1*11:01	183	0.0241	218	0.00727	491	1.68E-09	525	1.49E-10	358	57.2	4.01E-10	392	57.5	9.13E-12
DRB1*12:01	448	0.000138	473	5.46E-05	601	1.92E-08	619	7.31E-09	542	78.2	4.18E-12	566	78.1	4.78E-13
DRB3*02:02	48.3	0.284	58.6	0.214	352	2.24E-12	343	4.52E-11	205	33.7	1.19E-09	210	35	2.27E-09
**DQA1*05:05**	69.8	0.214	120	0.0374	505	1.65E-15	547	2.37E-17	297	42.2	2.17E-12	344	43.1	1.47E-15
*Haplotype: DRB1*04:[04]-DRB4*01-DQA1*03:01-DQB1*03:02*														
DRB1*04	-200	2.13E-07	-136	0.0011	-194	2.05E-05	-85.1	0.0806	-194	29.6	6.01E-11	-105	31.9	0.00106
**DRB1*04:04**	-283	4.89E-06	-191	0.00246	-296	0.000125	-184	0.018	-286	48.8	4.49E-09	-183	49.5	0.000229
DRB4*01	-154	1.77E-05	-91.7	0.0225	-196	6.99E-06	-72	0.141	-172	27.9	7.58E-10	-75.5	31.4	0.0161
DQA1*03:01	-188	3.20E-06	-118	0.00626	-165	0.000558	-50	0.322	-173	31.1	2.79E-08	-77.4	33	0.0191
DQB1*03:02	-199	1.48E-06	-133	0.0024	-147	0.00299	-38.7	0.453	-169	32.1	1.28E-07	-78	33.7	0.0208
*Haplotype: A*01:01-B*08:01-C*07:01-DRB1*03:01-DRB3*01:01-DQA1*05:01-DQB1*02:01*														
A*01:01	-22.2	0.571	4.06	0.918	-138	0.00521	-87.4	0.0776	-72.1	31.1	0.0203	-33.2	31.2	0.287
**B*08:01**	-79	0.0706	-26.5	0.553	-204	9.24E-05	-138	0.00869	-137	33.8	5.35E-05	-74.9	34.4	0.0293

C*07:01	-32.8	0.412	2.66	0.948	-148	0.0026	-108	0.0278	-84.1	31.3	0.00721	-43.4	31.6	0.169
DRB1*03:01	-56.9	0.193	10.7	0.815	-208	4.77E-05	-126	0.017	-130	33.5	0.000105	-52.8	34.8	0.129
DRB3*01:01	29.3	0.458	49.8	0.242	-124	0.00827	-156	0.00185	-46	30.4	0.131	-54.4	32.6	0.0952
DQA1*05:01	-41.6	0.329	29.9	0.505	-162	0.00114	-68	0.186	-101	32.6	0.00202	-16.6	34	0.626
DQB1*02:01	-65.6	0.131	-0.196	0.997	-227	7.87E-06	-144	0.00584	-145	33.2	1.30E-05	-68.9	34.6	0.0462
*Haplotype: DRB1*13:01-DQA1*01:03-DQB1*06:03*														
**DRB1*13:01**	110	0.044	188	0.000867	199	0.00104	254	3.53E-05	148	40.7	0.000268	218	41.6	1.74E-07
DQA1*01:03	89.2	0.0971	167	0.00276	193	0.00131	249	4.16E-05	134	40.2	0.00085	203	41.1	7.77E-07
DQB1*06:03	55	0.295	135	0.0136	198	0.001	260	2.14E-05	119	39.8	0.00274	192	40.8	2.66E-06
*Allele: DPB1*03:01*														
**DPB1*03:01**	-166	2.67E-05	-125	0.00178	-183	0.000391	-139	0.0074	-174	31.8	5.00E-08	-134	32.2	3.25E-05
*Allele: DPB1*04*														
DPB1*04:	183	9.18E-05	96.3	0.0514	73.4	0.198	-17.4	0.772	134	36.5	0.000238	44.5	38.6	0.249
*Haplotype: DRB1*08:01-DQA1*04:01-DQB1*04:02*														
**DRB1*08:01**	-182	0.00274	-97.5	0.119	-221	0.00463	-121	0.124	-195	48.7	5.93E-05	-99.1	49.7	0.0462
DQA1*04:01	-145	0.0147	-59.8	0.329	-220	0.00386	-116	0.131	-176	47.5	0.00021	-78.8	48.6	0.105
DQB1*04:02	-133	0.025	-45.7	0.454	-192	0.0116	-86.1	0.264	-157	47.4	0.000952	-56.8	48.4	0.241
*Allele: DRB1*11:04 [Controls only]*														
DRB1*11:04*	-146	0.302	-196	0.187	696	2.96E-05	348	0.0417	276	109	0.0113	66.3	113	0.556

The table lists HLA allele variants and their corresponding haplotypes associated to anti-EBNA-1 truncated IgG levels. Multivariable linear regression analyses were conducted both separately among MS cases and controls and also combined adjusting for MS affection status. All analyses were adjusted for sex, age at sampling, study type, and six PCA vectors. The top HLA alleles representing each haplotype are highlighted in bold and were all conditioned in the same model as shown above (*) to determine independent effects.

Table S5b. Summary of linear regression analyses examining the association between HLA variants and anti-EBNA-1 peptide IgG levels.

	MS Cases (n=7062)				Controls (n=6098)				All Subjects					
**Allele**	**β**	**P**	**β***	**P***	**β**	**P**	**β***	**P***	**β**	**SE**	**P**	**β***	**SE***	**P***
*Haplotype: A*03:01-B*07:02-C*07:02-DRB1*15:01-DRB5*01:01-DQA1*01:02-DQB1*06:02*														
A*03:01	50.2	1.09E-10	33.9	2.36E-05	39.1	0.000113	33	0.00181	45.4	6.24	3.57E-13	33	6.48	3.53E-07
B*07:02	38.9	4.98E-07	32.1	5.45E-05	56.9	3.48E-08	49.3	2.45E-06	46.2	6.27	1.95E-13	39.2	6.4	9.11E-10
C*07:02	37	1.50E-06	30.2	0.000127	59.7	4.37E-09	50.9	7.93E-07	46.5	6.21	7.67E-14	39.3	6.34	5.95E-10
DRB1*15:01	92.9	7.16E-34	86.4	5.80E-26	88.2	4.29E-18	92.5	1.97E-18	91.4	6.18	4.30E-49	89.9	6.52	6.34E-43
**DRB5*01:01**	93.8	1.62E-34	87.1	1.96E-26	88.4	3.67E-18	92.9	1.53E-18	92.1	6.18	8.20E-50	90.5	6.52	1.70E-43
DQA1*01:02	91.2	5.01E-31	84.3	2.06E-23	71.3	4.51E-14	79.9	1.37E-15	82.1	6.07	1.95E-41	82.7	6.48	4.33E-37
DQB1*06:02	94.5	4.11E-35	87.7	3.78E-27	86.8	2.21E-17	90.7	1.15E-17	91.7	6.18	2.03E-49	90	6.5	2.71E-43
*Haplotype: A*01:01-B*08:01-C*07:01-DRB1*03:01-DRB3*01:01-DQA1*05:01-DQB1*02:01*														
A*01:01	-23.2	0.00584	-32.7	0.000179	-41.6	7.12E-05	-28.3	0.00895	-31.1	6.62	2.61E-06	-30.2	6.86	1.10E-05
**B*08:01**	-54.9	4.99E-09	-47.2	1.11E-06	-71.5	9.39E-11	-55.6	9.60E-07	-62.3	7.19	4.56E-18	-50.7	7.41	8.16E-12
C*07:01	-31.3	0.000275	-26.6	0.00244	-55.4	9.04E-08	-43.4	3.78E-05	-42.1	6.66	2.62E-10	-33.6	6.8	7.56E-07
DRB1*03:01	-51.6	4.06E-08	-41.3	2.63E-05	-74.2	7.65E-12	-59.8	1.01E-07	-62.6	7.13	1.79E-18	-51.1	7.43	6.36E-12
DRB3*01:01	-25.6	0.00255	-19.2	0.045	-40.2	4.95E-05	-34.6	0.00198	-33	6.48	3.70E-07	-26.3	7.32	0.000323
DQA1*05:01	-49.7	5.46E-08	-39.5	3.78E-05	-64.4	1.06E-09	-53.9	7.82E-07	-57.1	6.94	2.17E-16	-47.7	7.23	4.36E-11
DQB1*02:01	-52.8	1.51E-08	-42.3	1.38E-05	-74.6	3.54E-12	-58.9	9.79E-08	-63.7	7.07	2.28E-19	-51.3	7.33	2.72E-12
*Haplotype: DRB1*11:01/12:01-DRB3*02:02-DQA1*05:05*														
DRB1*11:01	27.5	0.114	35	0.0424	90.5	1.53E-07	102	3.52E-09	63.2	12.2	2.28E-07	73.2	12.2	1.71E-09
DRB1*12:01	104	4.04E-05	114	4.76E-06	148	6.48E-11	153	9.99E-12	131	16.6	3.78E-15	139	16.5	5.02E-17
**DRB3*02:02**	12.3	0.206	15.3	0.127	81.5	1.76E-14	78	1.45E-12	47.9	7.17	2.44E-11	48.4	7.43	7.28E-11
DQA1*05:05	8.4	0.487	20.8	0.0884	104	1.28E-14	116	2.04E-17	57.6	9	1.60E-10	70.3	9.12	1.34E-14
*Haplotype: DRB1*07:01-DRB4*01-DQA1*02:01-DQB1*02:02/03:03*														

**DRB1*07:01**	-60.9	4.00E-07	-45.6	0.000218	-47.2	0.000222	-24.4	0.0658	-53.7	8.75	8.63E-10	-34.5	9.04	0.000136
DRB4*01	-44.9	5.49E-09	-17.3	0.0517	-50	5.78E-08	-13.4	0.205	-47.1	5.95	2.54E-15	-14.4	6.86	0.0355
DQA1*02:01	-64.3	9.58E-08	-48.5	9.39E-05	-44.3	0.000484	-21.6	0.101	-53.6	8.73	8.62E-10	-34	9.03	0.000169
DQB1*02:02	-58.4	5.99E-05	-40.1	0.00773	-57.1	0.000367	-31.8	0.0545	-57.8	10.8	8.57E-08	-35.8	11.1	0.00134
DQB1*03:03	-39.3	0.0224	-23.2	0.174	-6.46	0.687	11.1	0.488	-19.6	11.6	0.0928	-3.12	11.6	0.788
*Haplotype: DRB1*04:[04]-DRB4*01-DQA1*03:01-DQB1*03:02*														
DRB1*04	-38.5	3.40E-06	-21.4	0.0155	-40.2	3.13E-05	-16.6	0.113	-38.9	6.32	7.76E-10	-17.8	6.81	0.00902
**DRB1*04:04**	-56	2.57E-05	-32.8	0.0143	-56.6	0.000521	-30.1	0.0674	-56.1	10.4	6.90E-08	-31.3	10.5	0.00284
DRB4*01	-44.9	5.49E-09	-12.1	0.18	-50	5.78E-08	-12.7	0.245	-47.1	5.95	2.54E-15	-11.5	7	0.102
DQA1*03:01	-31.5	0.000291	-11	0.226	-29.4	0.00381	-3.8	0.723	-29.9	6.63	6.58E-06	-6.48	6.99	0.354
DQB1*03:02	-34.3	0.000118	-15.1	0.104	-26.4	0.0119	-1.36	0.902	-29.8	6.84	1.35E-05	-7.04	7.15	0.325
*Haplotype: DRB1*13:01-DQA1*01:03-DQB1*06:03*														
**DRB1*13:01**	26	0.0268	44	0.000274	60.6	2.29E-06	48.1	0.000218	42.8	8.67	8.23E-07	44	8.86	7.08E-07
DQA1*01:03	23.4	0.0429	40.9	0.000579	57.7	5.73E-06	45.8	0.000381	39.7	8.57	3.64E-06	41.1	8.74	2.57E-06
DQB1*06:03	19.1	0.0906	29.2	0.0113	57.2	7.22E-06	42.3	0.00102	37	8.48	1.28E-05	33.8	8.61	8.76E-05
*Allele: A*02:01*														
**A*02:01**	-34.2	7.13E-06	-43.8	8.52E-09	5.66	0.543	-3.15	0.734	-16.1	5.95	0.00675	-25	5.93	2.57E-05
*Allele: DPB1*03:01*														
**DPB1*03:01**	-54.6	1.12E-10	-47.8	1.20E-08	-51.5	2.53E-06	-44.2	4.76E-05	-53.2	6.77	4.04E-15	-46.6	6.72	4.62E-12
*Allele: DPB1*04*														
DPB1*04:	57	1.35E-08	19.3	0.0673	22.4	0.0639	-6.74	0.598	41.4	7.78	1.04E-07	8.43	8.2	0.304
*Allele: DPB1*11:01*														
**DPB1*11:01**	-117	7.66E-05	-92.2	0.00187	-71.3	0.0519	-49.5	0.177	-95.1	23.2	4.29E-05	-71.5	23.3	0.00215
*Allele: B*37:01 [Controls only]*														
**B*37:01**	66.6	7.70E-05	52.1	0.00168	-11.2	0.686	-15.3	0.575	42.4	14.8	0.00411	31.5	14.6	0.0302

The table lists HLA allele variants and their corresponding haplotypes associated to anti-EBNA-1 peptide IgG levels. Multivariable linear regression analyses were conducted both separately among MS cases and controls and also combined adjusting for MS affection status. All analyses were adjusted for sex, age at sampling, study type, and six PCA vectors. The top HLA alleles representing each haplotype are highlighted in bold and were all conditioned in the same model as shown above (*) to determine independent effects.

Table S5c. Summary of linear regression analyses examining the association between HLA variants and PepIndex.

	MS Cases (n=7062)				Controls (n=6098)				All Subjects					
**Allele**	**β**	**P**	**β***	**P***	**β**	**P**	**β***	**P***	**β**	**SE**	**P**	**β***	**SE***	**P***
*Haplotype: A*03:01-B*07:02-C*07:02-DRB1*15:01-DRB5*01:01-DQA1*01:02-DQB1*06:02*														
A*03:01	75.2	7.11E-13	53.2	8.03E-07	60.1	2.09E-07	45.7	0.00016	68.6	7.75	9.85E-19	49.6	8.04	6.75E-10
B*07:02	56.5	6.19E-08	47	9.09E-06	54.9	3.41E-06	44.7	0.000182	56	7.8	7.43E-13	46.1	7.89	5.38E-09
C*07:02	57.1	3.62E-08	47.1	7.45E-06	57.5	7.83E-07	46.4	8.28E-05	57.5	7.72	9.90E-14	46.9	7.82	2.01E-09
DRB1*15:01	92.7	3.33E-19	83.2	1.42E-14	70.5	1.49E-09	68.2	1.19E-08	83.7	7.71	2.51E-27	77.4	7.98	3.72E-22
**DRB5*01:01**	94	1.08E-19	84.3	5.91E-15	69.4	2.69E-09	67	2.12E-08	84	7.71	1.63E-27	77.5	7.98	2.95E-22
DQA1*01:02	92.2	4.21E-18	81.6	1.87E-13	54.5	4.74E-07	54.4	1.21E-06	74.5	7.57	8.78E-23	69	7.87	2.07E-18
DQB1*06:02	91.1	1.10E-18	81.6	2.77E-14	70.6	1.77E-09	68.5	1.19E-08	82.9	7.72	8.52E-27	76.7	7.96	6.99E-22
*Haplotype: A*01:01-B*08:01-C*07:01-DRB1*03:01-DRB3*01:01-DQA1*05:01-DQB1*02:01*														
A*01:01	-32.1	0.00455	-40.1	0.00054	-33.8	0.00487	-33.4	0.00672	-32.8	8.23	6.75E-05	-36.9	8.45	1.27E-05
**B*08:01**	-74.2	4.58E-09	-75.8	5.92E-09	-67.6	8.94E-08	-64	7.69E-07	-70.7	8.94	2.79E-15	-69.8	9.18	3.09E-14
C*07:01	-44	0.000144	-47.6	5.92E-05	-52.4	1.03E-05	-48.3	6.51E-05	-47.8	8.29	8.54E-09	-47.3	8.46	2.32E-08
DRB1*03:01	-73.4	6.82E-09	-71.2	7.48E-08	-71.4	8.87E-09	-64.3	5.80E-07	-72.6	8.87	3.01E-16	-67.8	9.22	2.13E-13
DRB3*01:01	-50.7	9.54E-06	-55.1	7.82E-06	-35	0.00202	-49	6.24E-05	-43.2	8.06	8.26E-08	-52.1	8.69	2.02E-09
DQA1*05:01	-75.2	1.04E-09	-73	1.39E-08	-66	4.54E-08	-60.4	1.39E-06	-70.9	8.63	2.19E-16	-67	8.98	8.55E-14
DQB1*02:01	-73.4	5.41E-09	-69.9	9.39E-08	-67.1	4.66E-08	-58.3	4.23E-06	-70.5	8.79	1.18E-15	-64	9.11	2.29E-12
*Haplotype: DRB1*07:01-DRB4*01-DQA1*02:01-DQB1*02:02/03:03*														
**DRB1*07:01**	-89.4	3.26E-08	-85.6	1.40E-07	-43.3	0.00309	-32.8	0.0275	-64.8	10.9	2.61E-09	-57.4	11	1.81E-07
DRB4*01	-38.2	0.000233	-21.7	0.0488	-34	0.00127	-15.9	0.164	-36.2	7.41	9.95E-07	-18.5	7.92	0.0193
DQA1*02:01	-90.4	2.62E-08	-86.3	1.27E-07	-41.1	0.00471	-31.3	0.0338	-63.8	10.9	4.37E-09	-56.4	11	2.77E-07
DQB1*02:02	-83.6	2.02E-05	-81.5	3.13E-05	-70.2	0.000132	-60.2	0.0011	-76.5	13.4	1.21E-08	-70	13.4	1.95E-07
DQB1*03:03	-87	0.000176	-72.3	0.00178	-14	0.446	-4.38	0.812	-43.5	14.5	0.00266	-31.9	14.5	0.0276
*Haplotype: DRB1*12:01-DRB3*02:02-DQA1*05:05*														

**DRB1*12:01**	56.5	0.0973	62.3	0.0651	94.6	0.000263	101	0.000104	80.3	20.7	0.000109	86	20.6	3.11E-05
DRB3*02:02	8.03	0.539	7.43	0.583	43.5	0.000357	38.1	0.00251	26.4	8.93	0.00316	23.6	9.24	0.0108
*Haplotype: DRB1*13:01-DQA1*01:03-DQB1*06:03*														
**DRB1*13:01**	19.5	0.219	40	0.013	54.1	0.000227	58.7	7.84E-05	37.3	10.8	0.00054	48.5	10.9	9.10E-06
DQA1*01:03	21.1	0.175	40.9	0.00991	51.8	0.000372	55.8	0.000156	36.8	10.7	0.000564	47.3	10.8	1.22E-05
DQB1*06:03	23	0.131	35.2	0.0219	48.3	0.000944	50.6	0.000589	35.5	10.6	0.000761	42.1	10.6	7.63E-05
*Allele: A*02:01*														
**A*02:01**	-52.3	3.44E-07	-65.2	2.47E-10	-25	0.0189	-34.2	0.00139	-39.8	7.39	7.46E-08	-50.7	7.41	8.08E-12
*Allele: DPB1*03:01*														
**DPB1*03:01**	-53.7	2.55E-06	-48.9	1.65E-05	-36.9	0.00319	-33.8	0.00671	-46.3	8.43	3.99E-08	-42.3	8.39	4.73E-07
*Allele: DPB1*04*														
DPB1*04:	51.7	0.000136	11.5	0.419	20.4	0.14	-2.13	0.885	37.4	9.68	0.000112	5.82	10.2	0.569
*Allele: DPA1*02:01*														
DPA1*02:01	-39.5	0.00297	-15.6	0.252	-27.3	0.0385	-8.49	0.531	-33.9	9.37	0.000295	-12.9	9.61	0.179

The table lists HLA allele variants and their corresponding haplotypes associated to the PepIndex. Multivariable linear regression analyses were conducted both separately among MS cases and controls and also combined adjusting for MS affection status. All analyses were adjusted for sex, age at sampling, study type, and six PCA vectors. The top HLA alleles representing each haplotype are highlighted in bold and were all conditioned in the same model as shown above (*) to determine independent effects.

Table S5d. Summary of linear regression analysis examining the association between HLA variants and anti-VCAp18 IgG levels.

	MS Cases (n=7062)				Controls (n=6098)				All Subjects					
**Allele**	**β**	**P**	**β**	**P**	**β**	**P**	**β**	**P**	**β**	**SE**	**P**	**β**	**SE**	**P**
*A*03:01-B*07:02-C*07:02-DRB1*15:01-DRB5*01:01-DQA1*01:02-DQB1*06:02*														
A*03:01	24	0.241	14.6	0.48	65.5	0.014	75	0.00564	41.3	16.4	0.0119	40.3	16.7	0.0155
B*07:02	119	4.41E-09	89.1	2.32E-05	144	1.19E-07	130	2.73E-06	127	16.5	1.43E-14	105	17	7.75E-10
**C*07:02**	123	1.23E-09	92.1	1.08E-05	136	3.56E-07	123	7.19E-06	126	16.3	1.19E-14	104	16.8	5.80E-10
DRB1*15:01	113	2.31E-08	84.6	7.49E-05	131	9.86E-07	120	1.23E-05	120	16.3	2.09E-13	101	17	3.11E-09
DRB5*01:01	115	1.25E-08	87.1	4.58E-05	135	4.64E-07	125	5.36E-06	123	16.3	5.06E-14	105	17	8.42E-10
DQA1*01:02	110	1.07E-07	80	0.000289	103	3.46E-05	92	0.000355	107	16	3.18E-11	86.4	16.8	2.87E-07
DQB1*06:02	115	1.11E-08	87.6	3.89E-05	132	1.04E-06	121	1.21E-05	122	16.4	1.00E-13	103	17	1.29E-09
*A*01:01-B*08:01-C*07:01-DRB1*03:01-DRB3*01:01-DQA1*05:01-DQB1*02:01*														
A*01:01	-25.1	0.256	-5.14	0.82	-88.2	0.00139	-75	0.00743	-52.6	17.4	0.00255	-35.8	17.7	0.0436
**B*08:01**	-137	3.10E-08	-118	3.94E-06	-89.2	0.00217	-78.2	0.00973	-112	18.9	2.94E-09	-96.8	19.7	8.84E-07
C*07:01	-104	4.37E-06	-80.8	0.000626	-74.1	0.00665	-60.2	0.0335	-88.5	17.5	4.52E-07	-68.9	18.3	0.000164
DRB1*03:01	-94	0.000144	-68.6	0.00815	-66.7	0.0195	-54.6	0.0691	-79.8	18.8	2.18E-05	-60.7	19.7	0.00211
DRB3*01:01	-73.7	0.000957	-34.1	0.143	-73.8	0.00464	-65.4	0.0159	-74.2	17.1	1.37E-05	-51.7	17.7	0.00359
DQA1*05:01	-88.6	0.000232	-62.1	0.0138	-62.5	0.0244	-49.7	0.0879	-75.4	18.3	3.76E-05	-55.6	19.2	0.00373
DQB1*02:01	-102	3.49E-05	-77.2	0.00273	-62.4	0.0273	-50	0.0932	-81.8	18.6	1.13E-05	-62.9	19.6	0.00133
*DRB1*04:04*														
**DRB1*04:04**	149	2.10E-05	143	4.32E-05	230	8.04E-08	222	2.31E-07	189	27.3	5.61E-12	181	27.4	3.59E-11
*DRB1*10:01*														
**DRB1*10:01**	-133	0.131	-125	0.156	-328	0.000279	-320	0.000389	-245	63	0.000101	-242	62.7	0.000112
*DRB1*09:01*														
**DRB1*09:01**	-11.7	0.878	-29.1	0.7	-299	2.06E-05	-303	1.51E-05	-185	51.1	0.000297	-195	50.9	0.000129
*C*12*														

**C*12**	-106	0.00484	-104	0.0057	-131	0.00631	-131	0.00642	-114	29.9	0.000134	-111	30	0.000206
*B*35:01-C*04:01*														
**B*35:01**	-91.6	0.00562	-88.5	0.00812	-95.3	0.0162	-96.1	0.0163	-92.3	25.6	0.00031	-92.3	25.8	0.000358
C*04:01	-66.7	0.0125	-57.8	0.0344	-61.4	0.0588	-57.9	0.0793	-63.6	20.8	0.00223	-57.3	21.2	0.00696
*Allele: DPA1*02:01 [Cases only]*														
**DPA1*02:01**	-104	6.64E-05	-72.1	0.00625	-30.7	0.311	-8.99	0.77	-67.4	19.8	0.000675	-41.7	20.1	0.0382
*Allele: DQA1*05:05 [Cases only]*														
**DQA1*05:05**	-125	7.77E-05	-119	0.000207	8.32	0.814	12.2	0.734	-54.8	23.7	0.0209	-48.7	23.9	0.0416
*Allele:DRB1*11:04 [Cases only]*														
DRB1*11:04	-302	0.000157	-219	0.00891	182	0.0496	194	0.0432	-61.3	61	0.315	-20.7	63.3	0.744

The table lists HLA allele variants and their corresponding haplotypes associated to anti-VCAp18 IgG levels. Multivariable linear regression analyses were conducted both separately among MS cases and controls and also combined adjusting for MS affection status. All analyses were adjusted for sex, age at sampling, study type, and six PCA vectors. The top HLA alleles representing each haplotype are highlighted in bold and were all conditioned in the same model as shown above (*) to determine independent effects.

Figure S6. Genome-wide associations to anti-EBV IgG levels and IM history.

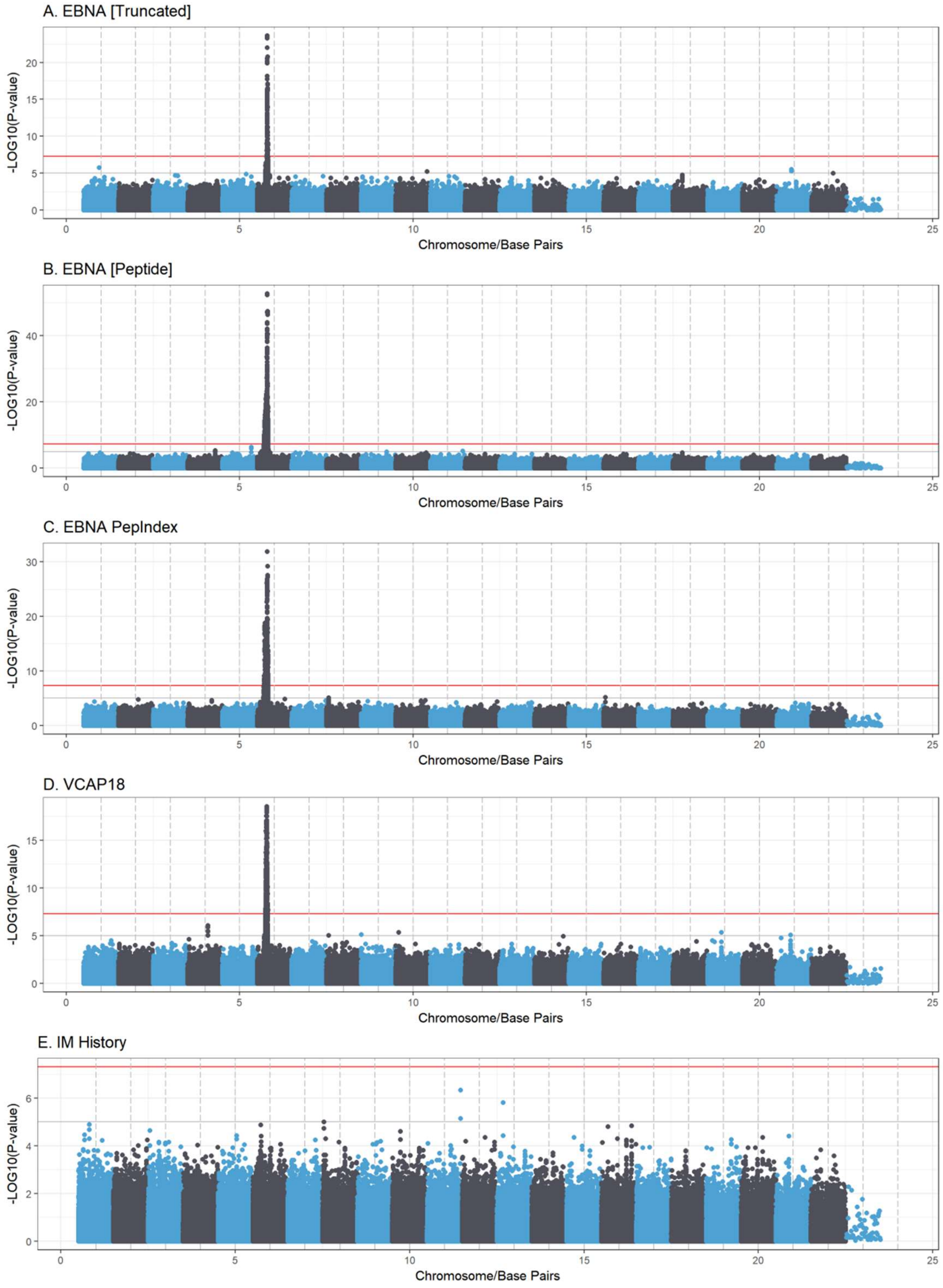

Manhattan plots summarizing multivariable linear regression analyses for examining genetic associations to anti-EBNA-1 and VCAp18 IgG levels along with IM history. Analyses were adjusted for sex, age at sampling, study type, MS status, and five PCA vectors. Genome-wide significance (red line) and nominal significance (grey line) are also plotted for reference.

Figure S7. Genome-wide associations to anti-EBV IgG levels and IM history, stratified by MS affection status.

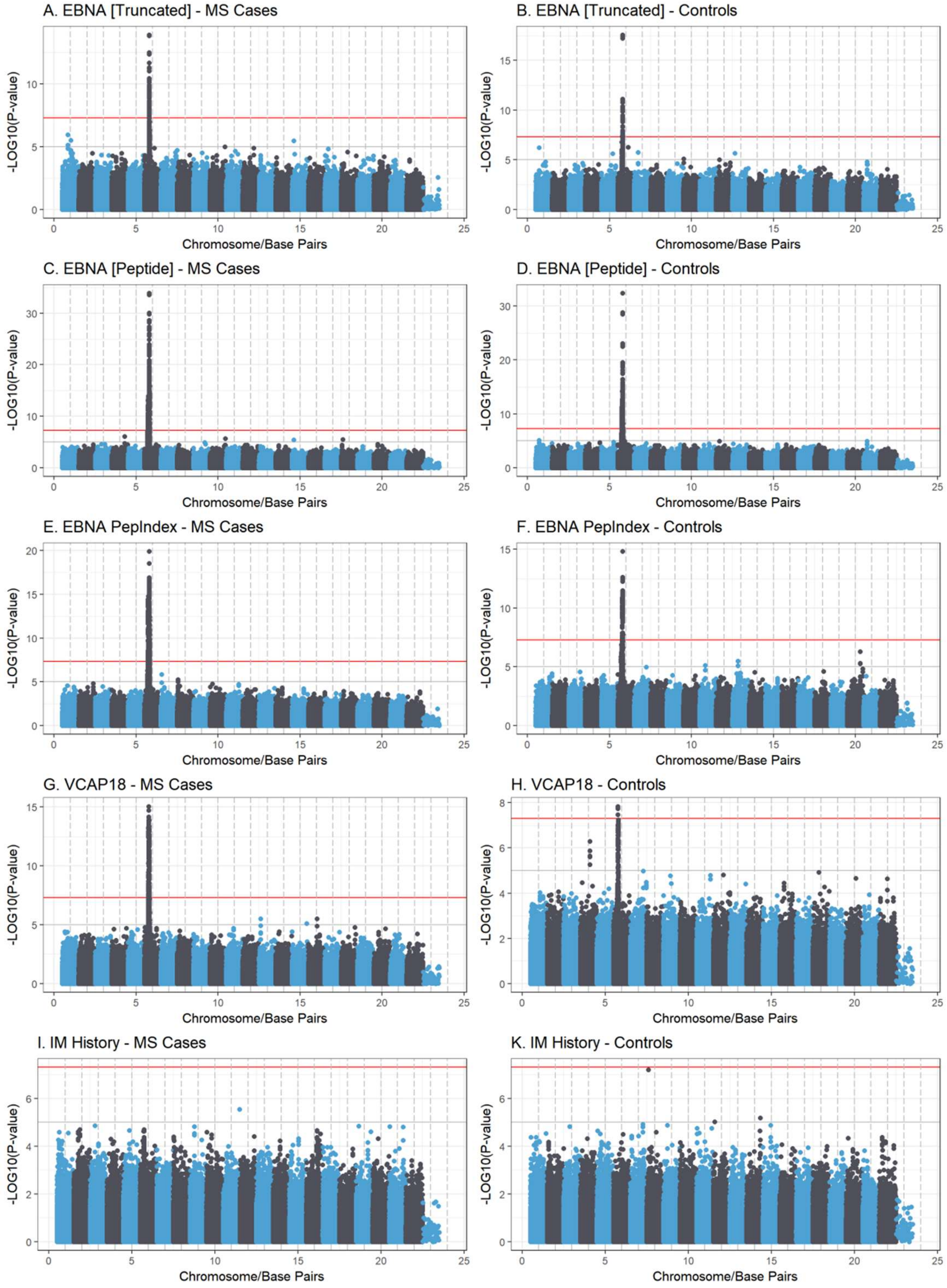

Similar to Figure S6, the Manhattan plots above summarize multivariable linear regression analyses for examining genetic associations to anti-EBNA-1 and VCAp18 IgG levels along with IM history within either MS cases or controls. Analyses were adjusted for sex, age at sampling, study type, and five PCA vectors. Genome-wide significance (red line) and nominal significance (grey line) are also plotted for reference.

Figure S8. Genome-wide associations to anti-EBV IgG levels conditioning for associated HLA allele variants.

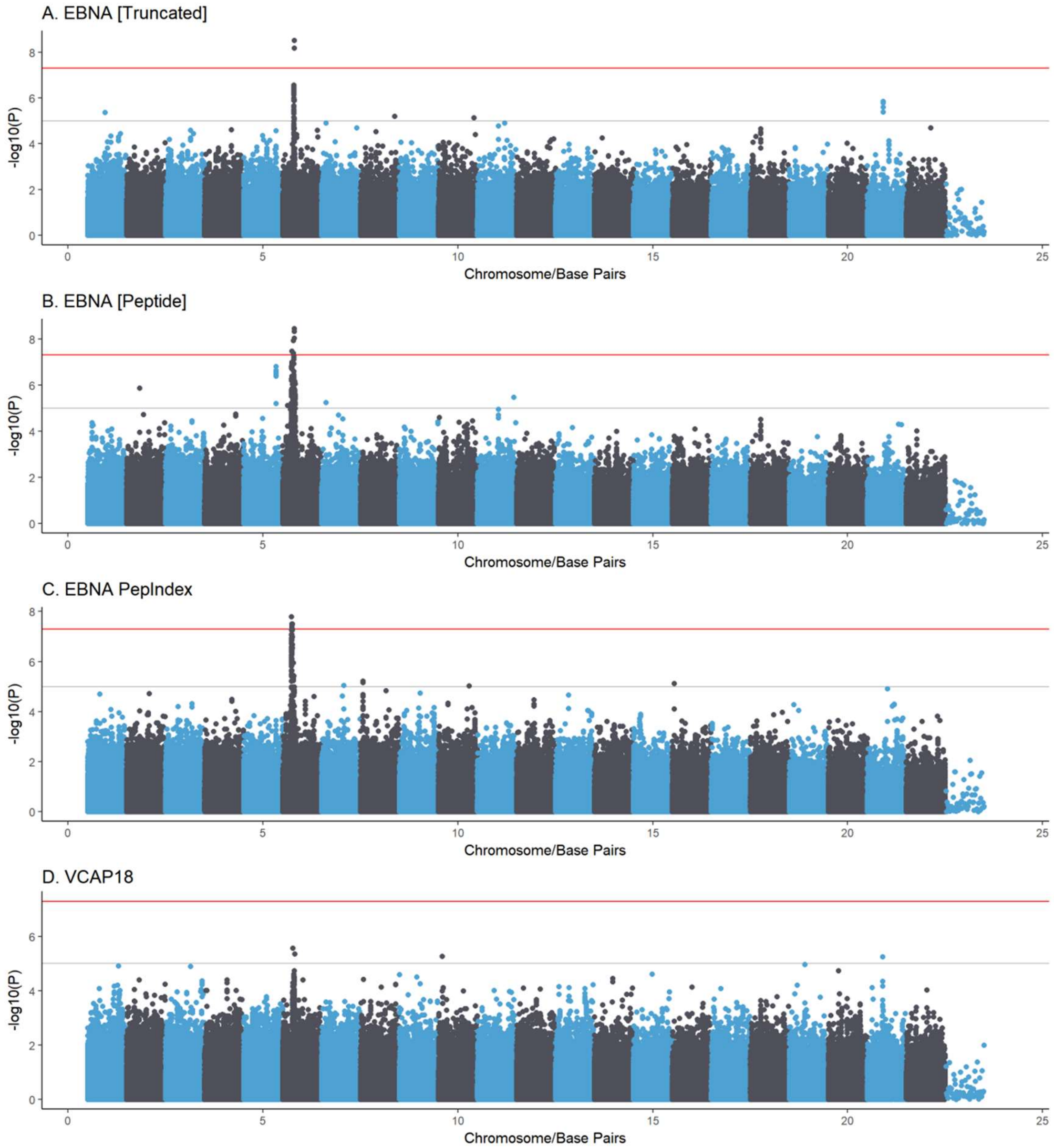

Manhattan plots summarizing multivariable linear regression analyses for examining genetic associations to anti-EBNA-1 and VCAp18 IgG levels conditioning for associated HLA haplotypes shown in Tables S5a-d. Analyses were adjusted for sex, age at sampling, study type, MS status, five PCA vectors, and carriage of relevant HLA alleles. Genome-wide significance (red line) and nominal significance (grey line) are also plotted for reference. After adjusting for associated HLA haplotypes, the association peak was significantly reduced (see Figure S6) but additional associations still remain in the HLA region.

Table S6. Residual HLA genetic associations to anti-EBV IgG levels below significance of P < $5x10^{-8}$.

CHR	BP	rsID	MA	β	SE	P
**Anti-EBNA-1 Truncated**						
6	32569691	RS477515	A	-173.2	29.19	3.08E-09
6	32570400	RS2516049	G	-169.4	29.19	6.64E-09
**Anti-EBNA-1 Peptide**						
6	32628428	RS2854275	A	-55.82	9.449	3.59E-09
6	32605884	RS2187668	A	-55.23	9.422	4.71E-09
6	32623223	RS9273327	C	-54.73	9.514	9.06E-09
6	31348077	RS9266669	A	-68.15	11.94	1.17E-08
6	30167294	RS9261558	G	31.01	5.616	3.45E-08
6	31434366	RS3094013	A	-89.71	16.33	4.03E-08

Genetic associations within the HLA region (Chr 6) from the GWAS findings illustrated in Figures S8 with significance below P<$5x10^{-8}$. No residual associations were observed with anti-VCAp18.

Table S7. Primary non-HLA genetic associations to anti-EBV IgG levels below nominal significance of $P < 10^{-5}$.

CHR	BP	rsID	MA	β	SE	P	Closest Gene
**Anti-EBNA-1 Truncated - All Subjects**							
1	99358358	rs984271	C	102	21.31	1.72E-06	PLPPR5
10	126681688	rs7097802	A	198.8	43.91	6.06E-06	CTBP2
21	29780523	rs2142272	G	176.5	38.73	5.21E-06	
21	29780950	rs9305370	A	177.7	38.55	4.08E-06	
21	29792308	rs2831792	A	185.8	39.84	3.15E-06	
21	29799861	rs2831800	G	181.7	39.31	3.85E-06	
22	39760883	rs739363	A	94.93	21.47	9.93E-06	
**Anti-EBNA-1 Truncated - MS Cases Only**							
1	70808932	rs6424385	A	-130.8	26.88	1.16E-06	ANKRD13C
1	70828536	rs4620474	G	-123.4	27.58	7.84E-06	HHLA3
1	116140239	rs6658990	A	-303	64.97	3.17E-06	
15	33669904	rs11633387	A	-246	52.87	3.34E-06	RYR3
**Anti-EBNA-1 Truncated - Controls Only**							
1	44989160	rs3842990	A	169.9	33.91	5.61E-07	RNF220
5	131395115	rs181781	A	-233.9	49.56	2.43E-06	
7	28189411	rs1635852	G	-155.6	32.47	1.71E-06	JAZF1
10	3076294	rs10903937	G	-179.9	40.21	7.84E-06	
12	21579080	rs7314170	A	-143.4	32.33	9.34E-06	
13	30486799	rs11617644	G	227.1	47.75	2.04E-06	
**Anti-EBNA-1 Peptide - All Subjects**							
4	157459397	rs6832535	A	-23.01	4.987	3.98E-06	
5	155763769	rs10061194	A	-39.93	8.142	9.52E-07	SGCD
5	155764666	rs10039835	A	-40.47	8.082	5.60E-07	SGCD
5	155779927	rs10059872	A	-40	8.103	8.05E-07	SGCD
5	155790030	rs10043401	C	-40.19	8.097	7.03E-07	SGCD
5	155800253	rs9313900	G	-41.36	8.129	3.67E-07	SGCD
11	128136389	rs565536	A	21.05	4.646	5.93E-06	
**Anti-EBNA-1 Peptide - MS Cases only**							
4	157459397	rs6832535	A	-31.32	6.35	8.29E-07	
10	129718838	rs12415045	A	28.28	5.951	2.05E-06	PTPRE
15	33669904	rs11633387	A	-52.65	11.37	3.74E-06	RYR3
18	6440339	rs7228477	A	-36.23	7.791	3.38E-06	
**Anti-EBNA-1 Peptide - Controls Only**							
1	44989160	rs3842990	A	32.09	7.183	8.08E-06	RNF220
**Anti-VCAp18 - All Subjects**							
4	111649869	rs10019689	A	86.27	19.44	9.15E-06	
4	111677722	rs10516563	C	90.56	19.55	3.67E-06	
4	111685081	rs4605724	A	91.09	19.56	3.22E-06	
4	111687514	rs6533527	A	94.31	19.46	1.28E-06	
4	111714889	rs2220427	A	95.8	19.39	7.89E-07	
8	4379269	rs10106920	A	-66.66	15.07	9.77E-06	
9	227980	rs12339394	G	106.4	23.76	7.56E-06	DOCK8
10	8220268	rs559872	A	-53.35	11.61	4.36E-06	
19	22503015	rs7252078	A	-54.01	11.74	4.30E-06	

21	28912400	rs8127160	A	56.1	12.62	8.86E-06	
**Anti-VCAp18 - MS Cases Only**							
13	26655996	rs17083171	A	168.6	36.08	3.03E-06	
15	97308588	rs2895446	G	-88.12	19.84	9.08E-06	
16	58288985	rs11076230	C	-93.88	20.06	2.92E-06	CCDC113
**Anti-VCAp18 - Controls Only**							
4	111649869	rs10019689	A	136.8	30.03	5.35E-06	
4	111677722	rs10516563	C	142.6	30.15	2.29E-06	
4	111714889	rs2220427	A	150.2	29.88	5.12E-07	
4	111685081	rs4605724	A	142.2	30.15	2.48E-06	
4	111687514	rs6533527	A	145.2	29.99	1.33E-06	

Genetic associations excluding those located in the HLA region from GWAS findings illustrated in Figures S6 and S7 with a significance below the nominal threshold of $P<10^{-5}$.

Table S8. Replication of previously established genetic associations to anti-EBNA-1 serology.

Antibody Measure	β	SE	L95	U95%	Stat	P
**rs11808092 - Chr1:93073228**						
EBNA-1 Truncated	-9.743	23.08	-54.98	35.49	-0.4221	0.6729
EBNA-1 Peptide	-2.85	4.923	-12.5	6.798	-0.579	0.5626
PepIndex	-1.323	6.152	-13.38	10.73	-0.2151	0.8297
VCAp18	-11.51	12.99	-36.98	13.95	-0.8861	0.3756
**rs669607 - Chr3:28071444**						
EBNA-1 Truncated	20.91	20.51	-19.28	61.1	1.02	0.308
EBNA-1 Peptide	4.359	4.374	-4.214	12.93	0.9966	0.319
PepIndex	3.4	5.462	-7.306	14.11	0.6225	0.5336
VCAp18	4.719	11.54	-17.91	27.35	0.4087	0.6828
**rs1962532 - Chr3:121751531 (LD proxy to rs2255214)**						
EBNA-1 Truncated	-23.05	20.53	-63.28	17.18	-1.123	0.2615
EBNA-1 Peptide	-9.539	4.378	-18.12	-0.9578	-2.179	0.02937
PepIndex	-12.14	5.47	-22.86	-1.419	-2.219	0.02648
VCAp18	-21.48	11.56	-44.12	1.172	-1.859	0.06311
**rs2104286 - Chr10:6099045**						
EBNA-1 Truncated	22.06	23.52	-24.05	68.16	0.9376	0.3484
EBNA-1 Peptide	1.855	5.014	-7.971	11.68	0.37	0.7114
PepIndex	-3.563	6.259	-15.83	8.704	-0.5693	0.5692
VCAp18	20.17	13.23	-5.762	46.11	1.525	0.1274

Four non-HLA genetic risk loci for association to anti-EBNA-1 antibody levels have been previously identified by Zhou and colleagues. Three of the four were genotyped on our Omniexpress chip and summary of association results are listed above for combined MS cases and controls. The SNP rs2255214 was not available on the same genotyping array but replaced with a linkage disequilibrium (LD) proxy SNP, rs1962532 (r^2=1, reference population of Utah residents from north and west Europe, CEU, and British in England and Scotland, GBR).

Table S9. Weights used for calculating anti-EBV IgG polygenetic risk score.

#	HLA allele	Effect [Weight]
**EBNA-1 Truncated**		
1	DQB1*06:02	289.61391
2	DQA1*05:05	344.04785
3	DRB1*13:01	217.54787
4	DPB1*03:01	-133.79423
5	DRB1*04:04	-182.60142
6	B*08:01	-74.92264
7	DRB1*08:01	-99.07416
**EBNA-1 Peptide**		
1	DRB5*01:01	90.47692
2	B*08:01	-50.71175
3	DRB3*02:02	48.43385
4	DPB1*03:01	-46.55396
5	DRB1*13:01	43.9705
6	DRB1*07:01	-34.48075
7	A*02:01	-24.96726
8	DRB1*04:04	-31.29756
9	DPB1*11:01	-71.4969
10	B*37:01	31.53993
**PepIndex**		
1	DRB5*01:01	77.52721
2	B*08:01	-69.77941
3	A*02:01	-50.71589
4	DRB1*07:01	-57.37183
5	DPB1*03:01	-42.26325
6	DRB1*13:01	48.54342
7	DRB1*12:01	86.0396
**VCAp18**		
1	C*07:02	99.69524
2	B*08:01	-100.7072
3	DRB1*04:04	177.62828
4	DRB1*10:01	-247.48361
5	DRB1*09:01	-197.89754
6	C*12	-111.36086
7	B*35:01	-95.44853
8	DPA1*02:01	-43.05994
9	DQA1*05:05	-50.91773

Conditioned effects of all independent HLA associations determined in Table S5a-d in the same linear regression model adjusting for sex, age at sampling, study type, and six PCA vectors.

Table S10a. Association between anti-EBV IgG levels and the individual MS risk HLA alleles.

	EBNA-1 [Trunc]		EBNA-1 [Pep]		PepIndex		VCAp18		IM History	
**HLA allele variants**	**β**	**P**	**β**	**P**	**β**	**P**	**β**	**P**	**β**	**P**
DRB1*15:01	312	**2.91E-47**	113	**9.82E-127**	113	**1.05E-83**	126	**8.07E-26**	0.01	**0.0102**
DRB1*15:01 HOM	308	**5.25E-05**	163	**2.01E-22**	200	**2.63E-22**	177	**2.71E-05**	0.04	**0.0412**
DRB1*03:01	1480	**6.30E-07**	661	**4.50E-24**	749	**1.15E-20**	818	**6.82E-07**	0.09	0.182
DRB1*03:01 HOM	-678	**6.98E-15**	-197	**7.75E-25**	-162	**5.76E-12**	-180	**0.000199**	-0.01	0.806
DRB1*13:03	-69	0.507	-23	0.307	-17	0.549	-61	0.288	-0.02	0.44
DRB1*08:01	-381	**0.000635**	-28	0.262	63	0.0365	148	**0.0163**	-0.03	0.293
DQB1*03:02	-597	**3.79E-09**	-115	**2.41E-07**	-39	0.151	55	0.324	0.01	0.569
A*02:01	82	0.166	92	**1.73E-12**	144	**1.62E-19**	70	**0.0327**	0.07	**9.97E-08**
A*02:01 HOM	231	0.107	196	**4.45E-10**	285	**1.70E-13**	259	**0.00106**	0.07	**0.0353**
B*44:02	-135	0.238	8	0.763	56	0.0688	17	0.79	0.01	0.702
B*38:01	296	0.06	79	**0.023**	60	0.16	168	0.0537	-0.01	0.798
B*55:01	512	**0.0275**	136	**0.00787**	135	**0.0316**	385	**0.00277**	-0.01	0.828
DQB103:01/02	803	**5.31E-08**	200	**7.50E-10**	149	**0.000187**	55	0.504	-0.01	0.808
DQA101:01*DRB115:01	-569	**0.000257**	-178	**2.02E-07**	-155	**0.00024**	-289	**0.000816**	-0.04	0.303

Association between individual MS-associated HLA genetic risks and measure of anti-EBV IgG or infectious mononucleosis (IM) history.

Table S10b. Association between anti-EBV IgG levels and the individual non-HLA MS risk SNPs.

	EBNA-1 [Trunc]		EBNA-1 [Pep]		PepIndex		VCAp18		IM History	
**SNP ID**	**β**	**P**	**β**	**P**	**β**	**P**	**β**	**P**	**β**	**P**
rs2255214	578	2.27E-05	161	9.13E-08	140	0.00015	251	0.000905	-0.02	0.579
rs11605422	846	0.000767	168	0.00241	60	0.377	323	0.0205	0.02	0.784
rs10245867	1653	0.000999	316	0.00422	70	0.605	532	0.0561	0.02	0.878
rs1870071	904	0.00148	192	0.00221	83	0.282	424	0.0071	0.1	0.145
rs883871	672	0.00187	117	0.0138	38	0.513	252	0.0353	0.05	0.36
rs32658	1219	0.00337	161	0.0779	-99	0.379	621	0.007	0.05	0.597
rs9909593	979	0.00349	185	0.0121	99	0.275	-74	0.69	0.05	0.541
rs17797448	627	0.00463	143	0.00342	83	0.165	224	0.0678	0.01	0.908
rs212397	357	0.00659	80	0.00578	45	0.209	86	0.235	0.07	0.0241
rs1801133	410	0.00725	89	0.00806	46	0.27	116	0.172	0.05	0.185
rs6738544	692	0.00885	139	0.017	48	0.503	322	0.0281	0	0.99
rs108990	660	0.0117	161	0.00512	106	0.135	326	0.0248	-0.03	0.573
rs12106877	-678	0.0127	-56	0.349	93	0.205	-370	0.0142	0.05	0.451
rs361725	360	0.0143	40	0.213	-28	0.484	-102	0.21	0.07	0.034
rs1151625	501	0.0146	124	0.00605	95	0.0854	133	0.243	-0.06	0.228
rs12087340	383	0.0154	93	0.00761	59	0.17	74	0.397	-0.01	0.778
rs4468527	-950	0.0171	-168	0.0556	-30	0.78	-266	0.227	0.1	0.291
rs58394161	601	0.0186	113	0.0437	30	0.667	345	0.0147	-0.05	0.43
rs1399180	417	0.0206	138	0.000487	113	0.0199	111	0.264	0.06	0.169
rs10951042	490	0.0209	105	0.0241	51	0.371	160	0.174	-0.01	0.792
rs756699	472	0.0223	105	0.0209	53	0.345	110	0.336	0.02	0.642
rs1323292	325	0.0248	90	0.00463	72	0.0671	9	0.913	0.03	0.288
chr12:9833628	307	0.0265	80	0.00871	74	0.0498	221	0.00399	-0.03	0.412
rs12708716	362	0.0276	94	0.00954	76	0.0859	277	0.00238	0.03	0.484
rs6928313	515	0.0292	204	8.98E-05	224	0.00045	-17	0.895	0.06	0.3

rs9878602	572	0.0302	196	0.000745	196	0.00593	282	0.0534	-0.05	0.414
rs767455	-222	0.0326	-57	0.0132	-37	0.19	-3	0.962	0.02	0.502
rs35716097	374	0.0335	107	0.0059	84	0.0796	227	0.0199	0.06	0.154
rs11897084	422	0.0348	95	0.0312	61	0.257	-100	0.369	0.04	0.412
rs28625973	457	0.0365	55	0.25	-18	0.762	218	0.0717	0.03	0.597
rs2445610	710	0.0369	221	0.00311	186	0.0435	167	0.376	0.01	0.915
rs9839229	819	0.0406	158	0.0722	56	0.603	51	0.82	0.05	0.554
rs224032	16286	0.0415	3243	0.0652	1570	0.468	5821	0.189	-1.09	0.552
rs249677	377	0.0461	73	0.0778	24	0.635	-58	0.582	0.05	0.222
rs1041796	440	0.0493	68	0.17	11	0.86	161	0.194	0.01	0.886
rs7665090	353	0.0545	110	0.0064	116	0.02	184	0.0709	0.04	0.3
rs1800693	102	0.0576	33	0.00546	31	0.0335	1	0.98	-0.01	0.679
rs59655222	346	0.0581	91	0.0232	51	0.305	168	0.0968	0.04	0.336
rs62626325	338	0.0609	108	0.00654	116	0.0173	-6	0.956	0.01	0.889
rs2248359	432	0.0617	123	0.0157	104	0.0978	279	0.0291	-0.09	0.0853
rs9562970	1213	0.0662	287	0.0484	177	0.321	301	0.41	0.35	0.0203
rs9843355	325	0.105	128	0.0038	141	0.00975	-56	0.617	-0.01	0.777
rs72922276	266	0.108	106	0.00367	103	0.0213	124	0.175	-0.02	0.559
rs9282641	465	0.108	158	0.0132	176	0.0244	256	0.11	0.02	0.749
rs506616	262	0.11	105	0.00366	112	0.012	208	0.0223	0.08	0.0371
rs4545915	479	0.112	165	0.013	136	0.0948	184	0.272	0.07	0.306
rs9913257	600	0.126	189	0.0288	179	0.0911	296	0.173	-0.07	0.453
rs117822168	313	0.131	129	0.00476	144	0.0102	177	0.123	-0.03	0.559
rs2413436	397	0.136	152	0.00951	165	0.0221	305	0.0389	0.03	0.649
rs6589706	336	0.137	111	0.0263	107	0.0799	192	0.126	-0.05	0.336
rs8070345	228	0.146	91	0.00856	110	0.00964	89	0.305	0.01	0.753
rs16902700	241	0.146	81	0.0268	83	0.0646	29	0.751	0.08	0.0487
rs1059091	380	0.149	-41	0.482	-177	0.013	294	0.0441	-0.03	0.582
rs10152892	304	0.168	99	0.041	89	0.138	114	0.352	-0.03	0.562

rs3761959	237	0.168	62	0.104	47	0.308	-208	0.029	0	0.96
rs34536443	737	0.171	319	0.00721	365	0.0122	1227	3.93E-05	-0.18	0.145
rs1051738	382	0.171	132	0.0323	156	0.0395	457	0.00317	0.05	0.415
rs438613	169	0.172	61	0.0257	75	0.0253	49	0.478	0	0.933
rs9292776	-555	0.177	-63	0.487	73	0.513	-248	0.275	0.19	0.0453
rs11740512	185	0.179	78	0.00959	83	0.0254	6	0.939	-0.03	0.322
rs12695416	510	0.193	170	0.0492	152	0.151	55	0.801	0.07	0.45
rs8006649	463	0.207	104	0.198	40	0.687	441	0.0302	-0.07	0.405
rs10141746	684	0.246	351	0.00686	396	0.0132	963	0.00322	0.11	0.407
rs116970203	229	0.249	110	0.0122	131	0.0148	33	0.763	0.01	0.779
rs2957873	239	0.367	180	0.00198	261	0.000272	-105	0.476	0.06	0.288
rs12460421	354	0.385	34	0.705	-41	0.711	538	0.0171	0.04	0.671
rs12330493	191	0.416	53	0.302	53	0.407	190	0.145	0.12	0.0334
rs11567694	-141	0.438	53	0.181	147	0.00278	-122	0.225	0.04	0.288
rs8022092	148	0.446	12	0.784	-17	0.75	44	0.684	-0.1	0.0239
rs9591325	164	0.481	152	0.00297	233	0.000222	-20	0.879	-0.05	0.389
rs12928822	131	0.482	74	0.0727	99	0.0498	-6	0.954	-0.04	0.361
rs7014582	-476	0.538	182	0.284	423	0.0435	-743	0.0831	0.18	0.303
rs7690934	147	0.543	111	0.0372	161	0.0136	-144	0.281	-0.06	0.285
rs2104286	177	0.553	141	0.0318	230	0.00428	-131	0.428	-0.01	0.883
rs9321490	98	0.578	80	0.038	120	0.0114	44	0.654	0.04	0.344
rs12373588	105	0.597	86	0.0476	136	0.011	145	0.187	0.06	0.208
rs1014486	125	0.663	121	0.0562	181	0.0199	443	0.00546	-0.02	0.745
rs13551	126	0.691	207	0.00299	318	0.00021	59	0.738	-0.06	0.414
rs10914539	69	0.73	29	0.514	44	0.411	-68	0.537	0.14	0.002
rs3766374	-62	0.817	80	0.181	171	0.0194	488	0.00112	0.01	0.817
rs77958473	-111	0.825	21	0.853	71	0.601	183	0.51	0.25	0.0293
rs4409785	40	0.828	67	0.103	98	0.0518	22	0.83	0.1	0.0209
rs1335532	12	0.886	43	0.0232	74	0.00161	-5	0.91	0.01	0.678

rs75029101	-19	0.951	161	0.0153	269	0.000997	288	0.0862	0.06	0.385

Association between individual multiple sclerosis (MS) non-HLA genetic risk scores (GRS) and measure of anti-EBV IgG or infectious mononucleosis (IM) history. Only GRS's with at least one association with a significance, $P < 0.05$, are listed above.

Table S11. Bi-directions genetic risk score association for anti-EBV IgG levels and MS.

	β	P	β	P
**MS wGRS (All) between high and low EBV antibody status**				
	**MS Cases**		**Controls**	
EBNA-1 Truncated	73.1	0.00000406	54	0.00517
EBNA-1 Peptide	27.3	1.23E-15	23.8	5.82E-09
PepIndex	27.7	1.64E-09	26.1	2.28E-08
VCAp18	41.4	3.86E-06	36.8	6.24E-04
**MS wGRS (HLA) between high and low EBV antibody status**				
	**MS Cases**		**Controls**	
EBNA-1 Truncated	85.2	0.000179	31	0.299
EBNA-1 Peptide	48.1	6.02E-23	32.6	0.00000024
PepIndex	59.9	8.75E-20	45.1	3.76E-10
VCAp18	70.9	3.36E-08	64.9	0.0000927
**MS wGRS (non-HLA) between high and low EBV antibody status**				
	**MS Cases**		**Controls**	
EBNA-1 Truncated	54.4	0.00898	69.2	0.00582
EBNA-1 Peptide	6.6	0.141	17	0.00132
PepIndex	-2.49	0.68	12.1	0.0466
VCAp18	11.8	0.315	16.1	0.248
**EBV wGRS (HLA) between MS cases vs Controls**				
	**All individuals**			
EBNA-1 Truncated	64.4	1.82E-67	-	-
EBNA-1 Peptide	27.3	4.43E-127	-	-
PepIndex	28.8	1.87E-144	-	-
VCAp18	21.1	1.52E-28	-	-

Weighted genetic risk scores (GRS) for both MS and anti-EBV IgG levels to determine cross-association with the other measures. Results from multi-variable linear regression model adjusting for sex, age at sampling, and study type.

Figure S9. Association between the genetic risk for MS and antibody levels against different EBNA-1 peptide fragments.

A. GRS - All genetic risks

MS Cases

EBNA-1	B	SE	P
1-89	3.37	1.090	2.08e-03
61-90	2.70	1.080	1.29e-02
385-420	4.03	0.802	5.82e-07
402-502	4.85	1.150	2.94e-05
421-450	2.72	1.150	1.85e-02

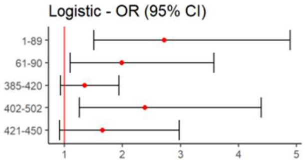

Controls

EBNA-1	B	SE	P
1-89	0.4300	0.940	0.64800
61-90	0.0597	0.881	0.94600
385-420	2.1600	0.737	0.00342
402-502	1.8600	1.350	0.17000
421-450	1.5000	1.100	0.17300

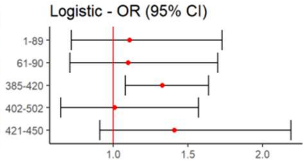

B. GRS - HLA only

MS Cases

EBNA-1	B	SE	P
1-89	4.47	1.51	3.12e-03
61-90	4.11	1.49	6.15e-03
385-420	4.85	1.10	1.20e-05
402-502	6.86	1.59	1.95e-05
421-450	3.13	1.59	4.97e-02

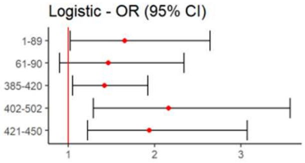

Controls

EBNA-1	B	SE	P
1-89	1.24	1.48	0.4050
61-90	1.02	1.39	0.4620
385-420	2.47	1.17	0.0348
402-502	2.07	2.14	0.3340
421-450	1.84	1.74	0.2910

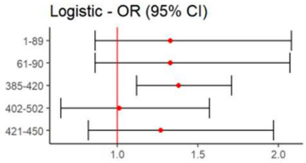

C. GRS - Non-HLA

MS Cases

EBNA-1	B	SE	P
1-89	1.710	1.42	0.2270
61-90	0.912	1.40	0.5160
385-420	2.540	1.07	0.0174
402-502	2.110	1.51	0.1630
421-450	1.790	1.49	0.2290

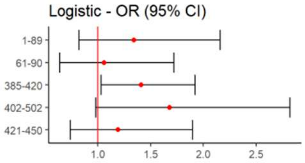

Controls

EBNA-1	B	SE	P
1-89	-0.116	1.24	0.9260
61-90	-0.613	1.16	0.5990
385-420	1.990	0.96	0.0387
402-502	1.800	1.79	0.3160
421-450	1.340	1.46	0.3600

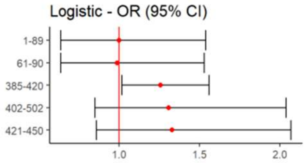

The above figures shows both [1] linear regression analyses of the association between continuous genetic risk for MS and IgG antibody levels against various anti-EBNA-1 peptide fragments (tables) and [2] logistic regression analyses examining the association between high/low genetic risk for MS and high/low IgG antibody status against said EBNA-1 peptide fragments (forest plot). Analyses were performed separately using either HLA genetic risks for MS (Panel B) or non-HLA (i.e., 200 risk loci[14], Panel C) and in combination (Panel A). In summary, antibody measures against the fragment 385-420, which is the primary EBNA-1 peptide measured in the main method and the main peptide associated with MS risk, is also associated with HLA and non-HLA GRS for MS in cases and controls.

Figure 2 HLA haplotypes associated with anti-EBV IgG levels. The above table summarizes all HLA alleles associated to anti-EBV IgG levels with significance of P<10-4, organized by corresponding haplotype (greyscale). Listed values are the beta [β] and significance [P] among all subjects adjusted for sex, age at sampling, multiple sclerosis affection status, and six PCA vectors. Color gradients were used to illustrate both significance level (darker=more significant) and type of effect: risk (red) and protective (blue). HLA-DRB4*01 (yellow) and its sub-types 01:01 and 01:03 are associated with the two haplotypes, DRB1*04:01 and DRB1*07:01.

**Allele**	EBNA1$_{Trunc}$ β	EBNA1$_{Trunc}$ *P*	EBNA1$_{Pep}$ β	EBNA1$_{Pep}$ *P*	PepIndex β	PepIndex *P*	VCAP β	VCAp *P*
A*03:01	37.1	0.206	45.4	3.57e-13	68.58	9.85e-19	41.34	0.0119
B*07:02	79.35	0.0071	46.15	1.95e-13	55.98	7.43e-13	126.99	1.43e-14
C*07:02	75.62	0.00961	46.49	7.67e-14	57.55	9.9e-14	126.18	1.19e-14
DRB1*15:01	262.35	2.48e-19	91.37	4.3e-49	83.7	2.51e-27	120.17	2.09e-13
DRB5*01:01	266.29	7.19e-20	92.06	8.2e-50	84.01	1.63e-27	123.24	5.06e-14
DQA1*01:02	241.55	3.36e-17	82.14	1.95e-41	74.55	8.78e-23	106.62	3.18e-11
DQB1*06:02	268.79	3.45e-20	91.75	2.03e-49	82.9	8.52e-27	121.87	1e-13
A*01:01	-72.1	0.0203	-31.11	2.61e-06	-32.81	6.75e-05	-52.56	0.00255
B*08:01	-136.56	5.35e-05	-62.35	4.56e-18	-70.71	2.79e-15	-112.4	2.94e-09
C*07:01	-84.14	0.00721	-42.15	2.62e-10	-47.76	8.54e-09	-88.52	4.52e-07
DRB1*03:01	-130.06	0.000105	-62.61	1.79e-18	-72.56	3.01e-16	-79.78	2.18e-05
DRB3*01:01	-46	0.131	-32.98	3.7e-07	-43.25	8.26e-08	-74.2	1.37e-05
DQA1*05:01	-100.73	0.00202	-57.05	2.17e-16	-70.94	2.19e-16	-75.38	3.76e-05
DQB1*02:01	-144.94	1.3e-05	-63.69	2.28e-19	-70.5	1.18e-15	-81.82	1.13e-05
DRB1*11:01	357.99	4.01e-10	63.16	2.28e-07	9.06	0.551	-18.46	0.565
DRB1*12:01	542.21	4.18e-12	131.05	3.78e-15	80.28	0.000109	-43.95	0.317
DRB3*02:02	204.86	1.19e-09	47.93	2.44e-11	26.36	0.00316	-48.44	0.0104
DQA1*05:05	296.82	2.17e-12	57.62	1.6e-10	19.57	0.0808	-54.75	0.0209
DRB1*13:01	148.37	0.000268	42.76	8.23e-07	37.33	0.00054	-9.6	0.674
DQA1*01:03	134.16	0.00085	39.69	3.64e-06	36.76	0.000564	-13.89	0.538
DQB1*06:03	119.3	0.00274	37.04	1.28e-05	35.54	0.000761	-7.37	0.741
DRB1*04	-194.04	6.01e-11	-38.87	7.76e-10	-14.63	0.0629	22.69	0.173
DRB1*04:01	-84.44	0.0162	-22.77	0.00234	-17.25	0.0637	-54.99	0.00519
DRB1*04:04	-286.42	4.49e-09	-56.15	6.9e-08	-18.57	0.152	188.53	5.61e-12
DQA1*03:01	-172.9	2.79e-08	-29.91	6.58e-06	-4.27	0.605	7.05	0.686
DQB1*03:02	-169.46	1.28e-07	-29.76	1.35e-05	-5.56	0.513	24.47	0.174
DRB1*07:01	-115.1	0.0051	-53.69	8.63e-10	-64.83	2.61e-09	-31.02	0.178
DQA1*02:01	-117.91	0.00406	-53.61	8.62e-10	-63.8	4.37e-09	-28.45	0.216
DQB1*02:02	-90.44	0.0745	-57.84	8.57e-08	-76.54	1.21e-08	-28.78	0.311
DQB1*03:03	13.3	0.808	-19.56	0.0928	-43.48	0.00266	-73.82	0.0159
DRB4*01	-171.97	7.58e-10	-47.1	2.54e-15	-36.25	9.95e-07	2.84	0.856
DRB4*01:01	-155.92	0.00309	-59.36	1.24e-07	-60.59	1.43e-05	-24.21	0.413
DRB4*01:03	-141.66	7.06e-07	-35	8.91e-09	-23.53	0.00189	8.17	0.61
DPB1*03:01	-173.51	5e-08	-53.25	4.04e-15	-46.32	3.99e-08	-14.79	0.407
A*02:01	38.69	0.166	-16.12	0.00675	-39.78	7.46e-08	-8.49	0.587
DPB1*04	134.23	0.000238	41.41	1.04e-07	37.4	0.000112	37.44	0.0675
DRB1*08:01	-195.46	5.93e-05	-20.1	0.0527	20.76	0.108	55.78	0.041
DQA1*04:01	-176.3	0.00021	-16.21	0.11	23.39	0.0635	60.5	0.0232
DQB1*04:02	-156.66	0.000952	-14.99	0.138	19.34	0.124	66.11	0.0129
DRB1*10:01	-248.23	0.0272	-50.89	0.0336	-14.17	0.634	-244.88	0.000101
DRB1*09:01	272.16	0.00284	40.28	0.0382	-8.38	0.729	-184.9	0.000297
DRB1*11:04	275.73	0.0113	38.63	0.0957	4.33	0.881	-61.28	0.315
B*35:01	-30.59	0.503	-5.25	0.589	-4.58	0.705	-92.26	0.00031
C*04:01	-13.61	0.714	-5.62	0.477	-8.27	0.4	-63.61	0.00223
C*12	7.52	0.888	12	0.291	16.85	0.233	-114.15	0.000134
DPA1*02:01	83.84	0.0178	-5.11	0.498	-33.92	0.000295	-67.38	0.000675
DPA1*11:01	-262.91	0.0159	-95.05	4.29e-05	-97.36	0.000753	-116.35	0.0569
B*37:01	148.6	0.032	42.37	0.00411	43.59	0.0176	77.68	0.0455
